# Differential microRNA profile underlies the divergent healing responses in skin and oral mucosal wounds

**DOI:** 10.1038/s41598-019-43682-w

**Published:** 2019-05-09

**Authors:** Alyne Simões, Lin Chen, Zujian Chen, Yan Zhao, Shang Gao, Phillip T. Marucha, Yang Dai, Luisa A. DiPietro, Xiaofeng Zhou

**Affiliations:** 1 Center for Wound Healing & Tissue Regeneration, Department of Periodontics, College of Dentistry, University of Illinois at Chicago, Chicago, IL USA; 2 Oral Biology Laboratory, Department of Biomaterials and Oral Biology, School of Dentistry, University of São Paulo, São Paulo, SP Brazil; 3 Center for Molecular Biology of Oral Diseases, Department of Periodontics, College of Dentistry, University of Illinois at Chicago, Chicago, IL USA; 4 Department of Bioengineering, College of Engineering, University of Illinois at Chicago, Chicago, IL USA; 5 College of Dentistry, Oregon Health and Sciences University, Portland, OR USA; 6 Graduate College, University of Illinois at Chicago, Chicago, IL USA; 7 UIC Cancer Center, University of Illinois at Chicago, Chicago, IL USA

**Keywords:** Epigenomics, Time series

## Abstract

Oral mucosal wounds heal faster than skin wounds, yet the role of microRNAs in this differential healing has never been examined. To delineate the role of microRNAs in this site-specific injury response, we first compared the microRNAome of uninjured skin and oral mucosa in mice. A total of 53 tissue-specific microRNAs for skin and oral mucosa epithelium were identified. The most striking difference was the high abundance of miR-10a/b in skin (accounting for 21.10% of the skin microRNAome) as compared to their low expression in oral mucosa (2.87%). We further examined the dynamic changes of microRNAome throughout the time course of skin and oral mucosal wound healing. More differentially expressed microRNAs were identified in skin wounds than oral wounds (200 and 33, respectively). More specifically, miR-10a/b was significantly down-regulated in skin but not oral wounds. In contrast, up-regulation of miR-21 was observed in both skin and oral wounds. The therapeutic potential of miR-10b and miR-21 in accelerating wound closure was demonstrated in *in vitro* assays and in a murine skin wound model. Thus, we provided the first site-specific microRNA profile of skin and oral mucosal wound healing, and demonstrate the feasibility of a microRNA-based therapy for promoting wound closure.

## Introduction

Impaired wound healing is a significant clinical problem, and affects approximately 6.5 million people in the United States. Wound healing is a multi-step process that in adults, generally ends with a scar. The attainment of a regenerative healing response remains one of the holy grails of the wound repair field. Although skin and oral mucosal wound healing proceed through the same stages of hemostasis, inflammation, proliferation, and remodeling, mucosal wounds demonstrate superior healing compared to skin wounds^1–4^. As such, comparative studies on paired skin and oral mucosal wounds represent a unique opportunity to reveal intrinsic regulators that orchestrate the differential healing process in adult tissues.

Studies in at least 3 different models (human, pig and mouse) now support the idea that rapid wound closure is a near universal feature of the oral cavity^1,5–7^. It has been suggested that the site-specific difference of wound healing is due to the environmental variations (temperature, saliva, microflora) and differences in inflammatory repertoire of two sites. However, skin transposed into oral cavity maintains its morphologic characteristics^8^, and may result in intraoral keloids^9^, suggesting that rapid wound healing in oral mucosa is likely to involve intrinsic characteristics of mucosal tissue rather than arising simply from environmental and inflammatory factors. Anatomic variation has also been suggested to play a role in the site-specific difference of wound healing. While structural differences do exist (e.g., cornified layer, hair follicles and sweat glands in skin; mucus layer, taste buds and salivary glands in oral mucosa), both oral mucosa and skin are stratified epithelium, and epithelial cells are critical to wound closure. Our recent studies demonstrate that epithelial cells from skin and mucosa exhibit functional differences in their response to injury^1,2,5,10^. For example, as compared to skin, isolated oral mucosal epithelial cells show increased migration and proliferation *in vitro*, and the *in vitro* results parallel the rapid wound closure seen in mucosa *in vivo*. Intrinsic differences in growth factor production and stem cell levels have all been suggested to support oral mucosal wound repair. These results suggest that the site-specific wound healing phenotype is stable and heritable, and involves intrinsic differences in epithelial cells and their response to a wound.

To date, available studies clearly demonstrate the complexity of wound healing at the genomic level. Our prior study provided the first dynamic transcriptome profiles of a paired oral mucosa and skin wound healing model, and showed that site-specific injury responses exist at each site^11^. More recent studies in humans also demonstrate that skin and oral mucosa differ both at baseline and throughout the time course of healing^12^. While these studies advance our understanding of wound healing, most prior efforts are focused on protein coding genes. Our knowledge of non-coding genes (e.g., microRNA) and their contribution to site differences in wound healing is limited. MicroRNAs have been shown to regulate many developmental, physiological and disease processes, including wound healing^13^. For example, hypoxia-induced miR-210 inhibits keratinocyte proliferation and impairs closure of ischemic wounds^14^, and miR-200b is involved in the induction of wound angiogenesis^15,16^. MiR-21, an oncomir identified in many cancers of epithelial origin, is also a wound healing regulator^17,18^. Our recent studies further illustrate that a change in the pattern of microRNAs is associated with specific phases of the wound healing process in skin^11,12^. For example, miR-99 family members (miR-99a, miR-99b, miR-100) are co-regulated during skin wound healing, and they regulate wound closure by targeting the IGF1R-AKT-mTOR signaling path. Together, these data suggest that microRNAs are fine-tuning regulators that contribute to the highly coordinated wound healing process. In this study, we explore the microRNA regulators associated with enhanced wound closure in oral mucosa. We also demonstrated the therapeutic potential of accelerating skin wound closure by manipulating the expression of selected microRNAs (miR-10b and miR-21) that are differentially expressed in mucosa and skin wounds. Our results provide a foundation for developing novel microRNA-based therapeutic approaches to promote skin wound closure and/or prevent chronic wounds.

## Results

### Baseline tissue specific miRNomes of skin and oral mucosa epithelium

**Figure 1:**
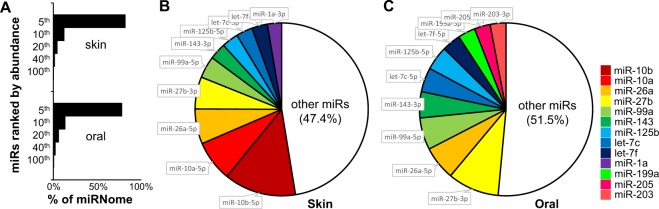
Baseline microRNAome landscape of skin and oral mucosa. MicroRNA profiling was performed on normal mouse skin and oral mucosa (hard palate) (n = 3 for both group). (**A**) The microRNA landscape was presented as histograms of percentage of microRNAome vs. microRNA species grouped by their abundance (top 5%, 6–10%, 11–20%, 21–40%, and 41–100%). The top 10 most abundant microRNAs account for 53% and 48% of the microRNAome in skin (**B**) and oral mucosa (palate) (**C**).

We first explored the differences in miRNome landscape of normal murine uninjured skin and oral mucosal epithelium (base line). Using miR-Seq analysis, a total of 601 unique microRNAs were mapped, in which the top 5% most abundant microRNA species account for 81.64% and 77.84% of the miRNomes of skin and oral mucosa epithelium, respectively (Fig. 1A). The top 10 most abundant microRNAs in skin and oral mucosa account for 53% and 48% of the miRNomes, respectively (Fig. 1B,C). The most striking difference was the high abundance of miR-10a and miR-10b in skin [accounting for (7.64 ± 1.01)% and (13.45 ± 2.14)% of the miRNome, respectively] as compared to their relative low expression in oral mucosa [(0.05 ± 0.02)% and (2.82 ± 0.83)%, p < 0.001 and p = 0.001, respectively] (Supplementary Table S1). Interestingly, the previously identified skin-specific microRNA (miR-203)^19,20^ was slightly more abundant in oral mucosa [(2.96% ± 0.27)%] than in skin [(1.93% ± 0.06)%, p = 0.042]. A total of 53 tissue-specific differentially expressed microRNAs were identified (p < 0.01) (Table 1, Supplementary Fig. 1, and Supplementary Table S2). Of these tissue-specific microRNAs, only miR-10a and miR-10b were among the top 10 most abundant microRNAs in the skin. None of the tissue specific microRNAs were among the top 10 most abundant microRNAs in oral mucosa.

**Table 1:** Differentially expressed microRNAs between skin and oral mucosa epithelium^a^.

miRNA	p-value	adj P^b^	log2 (skin/oral)	skin		oral	
				Mean	StDev	Mean	StDev
miR-378a-3p	1.29E-03	0.043	−1.33	15,167	183	6,013	400
miR-31-5p	1.85E-03	0.052	4.51	437	140	9,980	739
miR-10a-5p^c^	2.19E-03	0.054	−7.70	294,829	38,847	1,421	665
miR-10b-5p^c^	2.52E-03	0.059	−2.61	519,259	82,583	85,095	24,872
miR-133a-3p	4.19E-03	0.082	−3.82	55,766	31,546	3,956	1,681
miR-146b-5p	4.92E-03	0.083	−2.63	66,712	6,568	10,796	3,023
miR-126a-5p	6.54E-03	0.096	0.90	3,437	491	6,408	985
miR-34c-5p	7.34E-03	0.099	6.39	122	43	10,229	8,453
miR-30a-5p	8.14E-03	0.099	−0.71	47,463	4,893	29,008	1,047
miR-96-5p	8.19E-03	0.099	−0.79	11,975	1,738	6,919	917

^a^Cut-off p-value of 0.01 was used. Top 10 differentially expressed microRNAs that have the number of reads greater than the average (5458) of the dataset were listed. The complete list of 53 differentially expressed microRNAs was presented in Supplementary Table S1.

^b^Benjamini-Hochberg adjusted P-values were computed for multiple hypothesis testing.

^c^Both miR-10a-5p and miR-10b-5p were among the top 10 most abundant microRNAs in the skin.

### Injury induced miRNome changes in skin and oral mucosa epithelium

**Figure 2:**
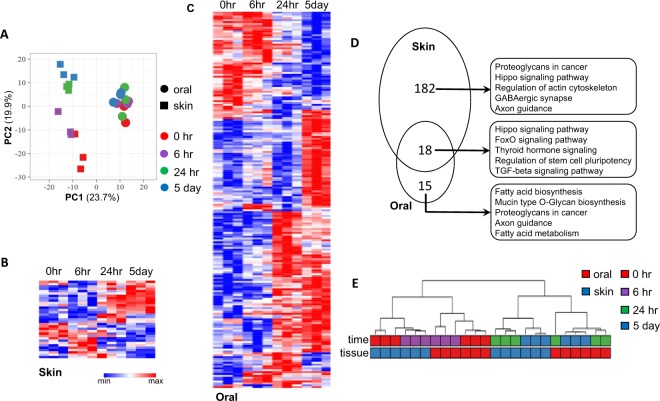
Comparison of microRNA expression during wound healing of skin and oral mucosal epithelium. Using a murine wound healing model of paired skin and oral mucosa wounds, microRNA profiling was performed on wound tissue samples acquired at 6 hr, 24 hr and 5 days post-wounding, and compared with unwounded baseline tissue samples (0 hr). (**A**) Principal component analysis (PCA) was performed on the whole dataset, and PC1 and PC2 explain 23.7% and 19.9% of the variance, respectively. PC1 can explain the variance associated with tissue specificity and PC2 appears to be associated with the variance related to wound healing time course. A total of 200 and 33 differentially expressed microRNAs (p < 0.01) were identified during oral mucosal (**B**) and skin epithelium (**C**) wound healing time course (0 hr, 6 hr, 24 hr and 5 days post-wounding), respectively. Among these microRNAs, 18 were concurrently differentially expressed (differentially expressed in both during skin and oral mucosa epithelium wound healing) (**D**, also see Supplementary Table S5). Common KEGG pathways regulated by differentially expressed microRNAs were predicted by DIANA-mirPath (v3.0)^38^ using microT-CDS^39,40^ as microRNA target prediction method (microT threshold = 0.8). Top 5 common KEGG pathways were presented. (**E**) A panel of 18-concurrently differentially expressed microRNAs was able to determine tissue specificity and wound healing time points based on hierarchical clustering analysis using on all samples (n = 24).

We further explored the differential miRNome response to injury using a murine wound healing model of paired skin and oral mucosa wounds, spanning all stages of the wound healing process (0 hr, 6 hr, 24 hr and 5 days post-wounding). Principal component analysis (PCA) was performed on the whole dataset, and PC1 and PC2 explain 23.7% and 19.9% of the variance, respectively (Fig. 2A). By applying a prediction ellipses probability of 0.95, PC1 can explain the variance associated with tissue specificity and PC2 appears to be associated with the variance related to wound healing time course (Supplementary Fig. S2). A total of 33 and 200 differentially expressed microRNAs were identified during the time course of oral mucosal and skin wound healing, respectively (Fig. 2A,B, Supplementary Tables S3 and S4). Among differentially expressed microRNAs that occur during wound healing, 18 of them were common among skin wound healing and oral mucosa wound healing (Fig. 2D, and Supplementary Table S5). Based on unsupervised learning method (hierarchical clustering analysis), this 18-concurrently wound healing associated microRNA signature was able to group/classify samples more preciously in terms of tissue specificity and wound healing time points as comparing to other microRNA signatures (e.g., 33-oral wound healing signature, 200-skin wound healing signature, 215-combined wound healing signature, and 53-baseline tissue specific microRNA signature) (Fig. 2E and Supplementary Fig. S3).

### Tissue specific miRNome landscape changes during the wound healing

**Figure 3:**
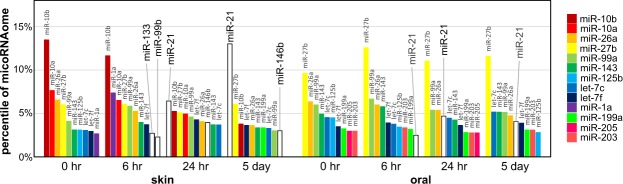
Injury induced microRNAome landscape changes during skin and oral mucosa wound healing. Changes in the levels of the top 10 most abundant microRNAs in skin and oral mucosa during the wound healing time course, expressed as % of the mRNAome.

To examine the tissue specific miRNome landscape changes during the wound healing, we calculated the % of the miRNome represented by each of the top 10 most abundant microRNAs during the time course of the skin and oral mucosa wound healing. As showed in Fig. 3 and Supplementary Table S1, for the top 10 most abundant microRNAs in skin, the most significant changes during wound healing were the down-regulation of miR-10a/b and the up-regulation of miR-21. Despite the down-regulation of miR-10a/b during skin wound healing, miR-10a/b levels were remarkably and significantly higher in skin wounds versus oral mucosal wounds at all time points. For the top 10 most abundant microRNAs in oral mucosa, the most significant change during wound healing was up-regulation of miR-21. Comparatively, the magnitude of miR-21 up-regulation was more pronounced in skin wounds than in oral mucosal wounds. At day 5 post-wounding, miR-21 became the most abundant microRNA in skin (13.0% of the miRNome), suggesting that miR-21 (a known oncomir that promotes cell proliferation and migration) plays an essential role in skin wound healing. In contrast, the up-regulation of miR-21 leveled off at 24 hr post-wounding in oral mucosal wounds (4.68% of the miRNome), suggesting that healing of oral mucosa is less dependent on miR-21 up-regulation, and consistent with the functional observation that oral mucosal tissue turns over much more rapidly than skin. In sum, these results suggested that miR-21 upregulation might be critical to skin repair.

**Figure 4:**
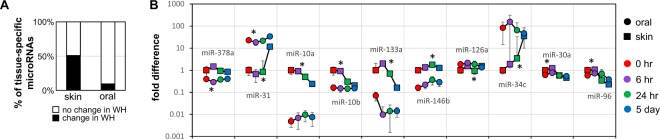
Injury induced changes of the baseline tissue specific microRNAome during skin and oral mucosa wound healing. (**A**) The percentage of those microRNAs that were identified as differentially expressed in normal uninjured tissue that also exhibit expressional changes during skin and oral mucosa during wound healing (WH). The difference is statistically significant (chi-square test p = 0.000003). (**B**) Changes in the levels of the top 10 tissue specific microRNAs between skin and oral mucosa during wound healing time course (*p < 0.01).

To explore the tissue specific miRNome landscape changes during the wound healing, we examined the changes of the baseline tissue-specific microRNAs during the skin and oral mucosa wound healing. As showed in Fig. 4A, of the 53-baseline tissue-specific differentially expressed microRNAs, 27 (50.9%) were differentially expressed during skin wound healing, and only 5 (9.4%) were differentially expressed during oral mucosa wound healing (chi-square test p = 0.000003). As showed in Fig. 4B and Supplementary Table S6, of the top 10- baseline tissue-specific differentially expressed microRNAs, 9 were differentially expressed during skin wound healing, and 2 were differentially expressed during oral mucosa wound healing.

### Effects of miR-21 and miR-10b on wound closure

**Figure 5:**
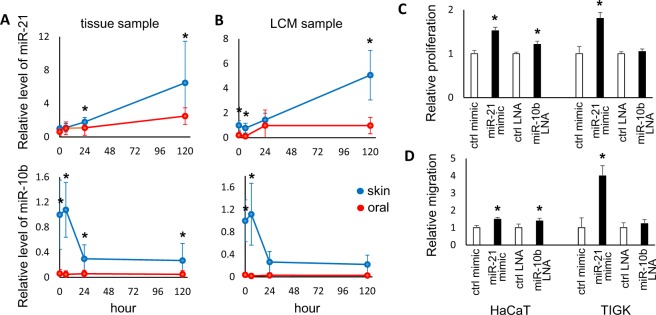
miR-21 and miR-10b: Differential expression in wounds and effect on proliferation and cell migration. (**A**) The wound tissue samples were procured from the paired skin and oral mucosa murine wound healing model, at 0 hr, 6 hr, 24 hr and 5 days post-wounding (n ≥ 6). The levels of miR-21 and miR-10b were assessed by TaqMan assay-based real time PCR quantification. Statistical significant changes in miR-21 and miR-10b levels were observed between skin and oral mucosal wounds during the time course (two-way ANOVA test p = 0.0054 and p < 0.0001, respectively). *Indicates statistical significant difference at specific time point (multiple t-test p < 0.05). (**B**) The LCM isolated epithelial cells from the wound edges were procured from the paired skin and oral mucosa murine wound healing model, at 0 hr, 6 hr, 24 hr and 5 days post-wounding (n = 4). The levels of miR-21 and miR-10b were assessed by TaqMan assay-based real time PCR quantification. Statistical significant changes in miR-21 and miR-10b levels were observed between skin and oral mucosal epithelial cells during the time course (two-way ANOVA test p = 0.0001 and p < 0.0001, respectively). *Indicates multiple t-test p < 0.05. *Indicates statistical significant difference at specific time point (multiple t-test p < 0.05). Statistical analyses were presented in Supplementary Table S7. HaCaT and TIGK cells were transfected with either negative control microRNA, miR-21 mimic, negative control LNA, or LNA inhibitor against miR-10b. Proliferation (**C**) and cell migration (**D**) were measured as described in the Material and Methods section. Data represents at least 3 independent triplicate experiments with similar results (*p < 0.05).

To confirm the differential expression of miR-21 and miR-10b, TaqMan assay-based validation was performed in additional wound tissue samples (n ≥ 6) and the epithelial cells from the wound edges isolated by laser capture microdissection (LCM) (n = 4). As showed in Fig. 5A,B, statistically significant up-regulation of miR-21 was observed in skin wound healing and in oral mucosal wound healing, although to a lesser extent in mucosa. In contrast, a statistically significant down-regulation of miR-10b was observed in skin wound healing. No change in miR-10b was seen over the course of oral mucosal wound healing, and of note, even normal oral mucosa had little miR-10b expression. Statistical analyses were presented in Supplementary Table S7.

We then tested the functional effects of miR-21 and miR-10b on wound healing using *in vitro* proliferation assays and migration assays. Since our expression data suggested that miR-21 might be a critical enhancer of wound healing, coupled with its well-established functions in proliferation and cell migration^17,18,21^, our approach was to increase miR-21 levels. In contrast, since miR-10b was seen to be expressed only in skin but not oral mucosal wounds, experiments were performed to inhibit miR-10b expression in skin. As showed in Fig. 5C, when the skin epithelial cell line (HaCaT) and the oral mucosal epithelial cell line (TIGK) were transiently transfected with the miR-21 mimic, enhanced proliferation was observed both HaCaT and TIGK as compared to cells transfected with control mimic. In contrast, locked nucleic acid (LNA)-mediated miR-10b knock-down resulted in enhanced proliferation in HaCaT, but not TIGK. Similarly, ectopic transfection of miR-21 enhanced the cell migration in both HaCaT and TIGK, while LNA-mediated miR-10b knock-down resulted in enhanced cell migration in HaCaT but not TIGK (Fig. 5D). While minor differences in response to miR-21 and miR-10b treatments were observed between these two cell lines (possibly due to the differences in cell origins and culture conditions), the combined results suggest that miR-21 facilitates rapid repair, while miR-10b inhibits it.

**Figure 6:**
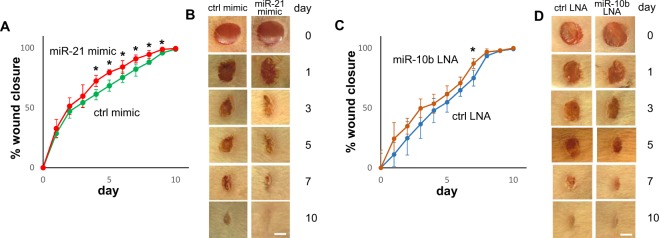
Effect of miR-21 and miR-10 on wound closure. (**A**) Mouse skin wounds (n = 6) were treated with miR-21 mimic or negative control mimic at the time of injury, and wound closure was measured for 10 days. Statistical significant changes in wound closure were observed between wounds treated with miR-21 mimic and wounds treated with negative control mimic (two-way ANOVA test p < 0.0001). *Indicates statistical significant difference at specific time point (multiple t-test p < 0.05). Statistical analyses were presented in Supplementary Table S8. (**B**) Representative photomicrographs of microRNA mimic treated wounds taken at the time points indicated. (**C**) Mouse skin wounds (n = 6) were treated with LNA inhibitor for miR-10b or negative control LNA at the time of injury, and wound closure was measured for 10 days. Statistical significant changes in wound closure were observed between wounds treated with miR-10b LNA inhibitor and wounds treated with negative control LNA (two-way ANOVA test p = 0.0001). *Indicates statistical significant difference at specific time point (multiple t-test p < 0.05). Statistical analyses were presented in Supplementary Table S8. (**D**) Representative photomicrographs of LNA treated wounds taken at the time points indicated. Scale bar = 2 mm.

To assess the therapeutic potential of promoting wound closure *in vivo*, levels of miR-21 and miR-10b were manipulated in a mouse skin wound healing model. An animal origin-free lipid nanoparticle *in vivo* delivery system was used to introduce the miR-21 mimic or a LNA inhibitor of miR-10b into the wounds. The effectiveness of the microRNA mimic and LNA inhibitor mediated up-regulation of miR-21, and the knock-down of miR-10b were confirmed by TaqMan assays performed on the wound tissue samples (Supplementary Fig. 4). As showed in Fig. 6A,B, a single dose of miR-21 mimic treatment led to statistical significant acceleration of wound closure, as compared to wounds treated with negative control mimic. Similarly, a statistically significant acceleration of closure was observed in wounds treated with the miR-10b LNA inhibitor as compared to wounds treated with negative control LNA (Fig. 6C,D). Statistical analyses were presented in Supplementary Table S8.

## Discussion

This is the first systemic, comprehensive and dynamic comparison of site-specific microRNAome profiles in corresponding skin and oral mucosal wounds. Together with our previous study that established the site-specific transcriptome of matching skin and mucosal wounds^11^, our results demonstrate striking differences in the transcribed genome (both transcriptome and microRNAome) of oral mucosal and skin wounds. Along with studies by others^12,22^, our results suggest that the differences in the genetic and epigenetic responses to injury in skin and mucosa contribute to the divergent wound healing outcomes. These findings at a genetic level are in agreements with previous observations suggesting that intrinsic differences, such as growth factor production, stem cell levels, and cellular proliferation capacity contribute to the superior repair in oral mucosa^23^.

The current study also establishes the baseline differences of the site-specific microRNAome for normal skin and oral mucosal epithelium. In tandem with what has been described at the transcriptome and cellular level, it appears that some of the divergence in wound healing process involves tissue-specific (and/or anatomic) variation that occurs between two sites even prior to injury. In terms of the microRNAome, miR-10a/b are both highly expressed in skin epithelium with minimal expression in oral mucosa and wounds. A significant down-regulation of miR-10a/b occurs in skin wounds and this down-regulation seems to support the healing of skin wounds. Oral mucosa and skin are both stratified epithelium, yet these tissues do exhibit multiple microscopic and anatomic differences. Certainly, these various differences may account for some of the differential transcriptional events (for both coding genes and microRNA genes) that are observed during the injury response at the two sites. However, studies in palate, buccal mucosa, and tongue models all demonstrate that oral mucosa, regardless of specific anatomical features, heals more rapidly than skin.^1,7,11,12^ Thus, the improved response to injury seen in oral mucosa seems to partially depend on tissue-specific elements (and/or adnexal structures).

One of our important observations is that more microRNA genes were differentially expressed in skin wounds than in oral mucosal wounds over time. This result implies that, as compared to skin, the oral mucosa has an intrinsic and refined genetic response that accelerates repair. This result is in keeping with our prior study that demonstrated that nearly twice as many protein coding genes are differentially expressed in the skin wounds than in mucosal wounds^11^. One possible explanation for the apparent differences in the transcribed genome (for both transcriptome and microRNAome) could be that the mucosa, being “preactivated” would not require a significant increase in the expression of genes during the healing process. For example, 8.7-fold upregulation of miR-21 expression was observed in skin wound healing, while 3.1-fold miR-21 upregulation was observed in oral mucosa wound healing. This idea has been recently proposed in a study that examined the genomic response in human buccal mucosal and skin wounds^12^. However, since this prior study examined only two time points, both of which were after the point of wound closure, a more complete analysis will be needed. Another possible reason for the larger genomic response in skin wounds might be that some of the highly expressed skin-specific microRNAs may act as suppressers of injury response. For example, miR-10a/b is high abundant in skin epithelium (accounts for 21.1% of the skin miRNome) as compare to oral mucosa (less than 3% of the oral mucosa miRNome). Both miR-10a and miR-10b exhibited similar expressional pattern changes in our paired wound healing model (50% and 70% reduction in skin wounds at 24 hours, respectively, and no change in oral mucosal wounds). Since they share identical seed-regions, it is reasonable to assume that they have similar target genes and similar biological functions. The miR-10 gene family is known for its co-evolution with HOX genes, its localization within the HOX gene clusters, and its ability to regulate HOX genes by targeting HOX transcripts^24^. MiR-10 belongs to miR-10/100 super-family. MiR-100 is believed to be the most ancient animal microRNA and to have arisen in a common ancestor of eumetazoa approximately 650 million years ago. At the root of the bilaterian lineage, duplications of miR-100 gave rise to miR-10^25^. Our previous studies demonstrated that miR-100 consistently downregulates during skin wound healing and targets HOXA1^26,27^, a known proto-oncogene which may play an important role in regulating epithelial cell proliferation and migration in physiological processes (i.e., skin wound healing). Since the miR-10/100 family is evolutionarily ancient and highly conserved, miR-10/100-mediated suppression of HOX gene family appears to be an evolutionary conserved function to maintain normal epithelium physiology of the skin. To activate the injury-induced genomic response program, these suppressers (e.g., miR-10/100) need to be downregulated in the skin, a fact that adds complexity and additional steps as compared to oral mucosa. Overall, the differences in the baseline programs may derive from the functional observation that oral mucosal tissue turns over much more rapidly than skin^28,29^. The rapid turnover of oral mucosal has been suggested to support the need for the oral mucosa to repair frequent small injuries and to regularly shed the microbiome.

We further tested the therapeutic potential of accelerating skin wound closure by manipulating specific microRNAs in skin wounds. MiR-21 and member of miR-10 family were chosen as our targets since they represent microRNAs that show differential response to injury and tissue-specific microRNAs that are differentially expressed at baseline. For the miR-10 family, we chose miR-10b over miR-10a based on statistical significance and fold changes (p = 0.0067 vs. p = 0.0096 and 70% vs. 50% reduction in skin wounds at 24 hours, Supplementary Table S4). Both miR-21 and miR-10b sequences are highly conserved across the species (e.g., identical mature microRNA sequences in human and mouse). Using a novel animal origin-free lipid nanoparticle-based *in vivo* delivery system, we were able to achieve miR-21 upregulation and miR-10b down-regulation in skin wounds. While the end points are similar (all wounds closed by day 10 in our model system), statistical significant acceleration of closure was observed in both types of microRNA treated skin wounds. The time points most affected by the introduction of more miR-21 were those surrounding the midpoint of healing. In contrast, miR-10b knock-down appears to affect wound closure both at early stage and at a later time point. However, the early stage change is not statistically significant. The differential timing of the effects may derive from the functionality of each microRNA and relate to the downstream gene families that each microRNA influences. It is worth noting that miR-21 is a well-established oncomir for many cancers of epithelial origin, and is a known wound healing regulator that controls multiple aspects of healing process in both human and mouse studies, such as promoting proliferation, cell migration and enhance stem cell survival^17,18,21,30^. Our results confirmed the previous observed effect of miR-21 on skin wound healing, where antagomir-mediated knockdown of miR-21 delayed the closure of skin wounds^17^. The functional role of miR-10 family members in wound healing is not entirely clear. Our results, together with previous observations of consistently high expression of miR-10a/b in mouse fetus and adult skin tissues^21,31,32^, suggest that miR-10a/b is a skin specific microRNA. A recent study on diabetic wound healing showed that the down-regulation of miR-10a/b was partially blocked in diabetic wounds as compared to normal skin wounds^21^, which may contribute to the delayed healing of diabetic wounds. This report, together with our observed difference of miR-10a/b level in skin and oral mucosa wounds and its effect in skin wound healing, suggest that the downregulation of miR-10 family members is an essential step in skin wound healing.

Taken together, our data identified a set of microRNAs that contribute to the differential response to injury in skin and oral mucosa. These include tissue-specific microRNAs that are differentially expressed at baseline (such as miR-10a/b), and microRNAs that show a differential response to injury (such as miR-21). The use of microRNAs to treat wounds is currently under investigation by several groups, and it holds promise as a novel and relatively inexpensive future treatment. As compared to other treatment modalities, microRNA treatment modifies multiple pathways at once, a feature that is attractive due to the complexity of wound pathology. The novelty of the current study is the discovery process, as the comparison of oral mucosal to skin wounds allowed the identification of novel microRNA targets. Our functional studies showed that the manipulation of these microRNAs in skin wounds *in vivo* can accelerate wound closure. This acceleration was shown in normally healing wounds, a model that exhibits rapid repair even in the naive state. The results suggest the feasibility of a microRNA-based therapeutic approach to promote wound closure and/or prevent chronic wounds. Additional studies with more time points, larger sample size and with impaired wound models (diabetic, chronic and/or infected wounds) will be needed to fully explore the translational value of our findings.

## Materials and Methods

### Animals and wound models

The experimental animal protocol was approved by the University of Illinois at Chicago Office of Animal Care and Institutional Biosafety Committee, and all procedures were performed in accordance with the National Institutes of Health (NIH) Guide for the Care and Use of Laboratory Animals. All animals were female 8-10-week-old Balb/c mice purchased from the Jackson Laboratory (Bar Harbor, ME) and were housed in groups of three to five in a temperature-controlled environment (22 to 24 °C) on a 12-h: 12-h light-dark cycle and provided with food and water ad libitum. To compare wound healing in skin and oral mucosa, the previously established mouse excisional skin and oral mucosal wound healing models^1,2^ was adapted with modifications.

For microRNA profiling in normal skin and oral tissues, mice were anesthetized via intraperitoneal injection with ketamine (100 mg/kg) and xylazine (5 mg/kg) solution. The dorsal surface of each mouse was shaved using electric clippers. Full thickness skin samples were collected with a 5 mm skin biopsy punch (Acu Punch, Acuderm, Ft. Lauderdale, FL). Full thickness hard palate was removed from maxilla with a pair of forceps. Harvested skin and palate tissues were placed in RNAlater (Sigma-Aldrich, St. Louis, MO) and stored at −20 °C until use.

For microRNA profiling in wounded skin and oral tissues, mice were anesthetized, shaved, and cleaned thoroughly with 70% isopropyl alcohol. Two 1 cm long full thickness incisional wounds were made on the dorsal skin of mice (one on each side of the midline) with a pair of scissors and three 0.5 cm incisional wounds were created on the anterior of the hard palate with a scalpel blade surgical #15 (EXELBLADES, Paterson, NJ). At 6 h, 24 h and 5 days post-wounding, the animals were sacrificed and specimens were harvested, placed in RNAlater (Sigma-Aldrich, St. Louis, MO) and stored at −20 °C until use.

### *In vivo* treatment with microRNA mimics or LNA inhibitors

To examine the effect of microRNA mimics or locked nucleic acid (LNA) inhibitors on skin wound healing, female 8–10 weeks old Balb/c mice were anesthetized and the dorsal skin was prepared as described above. Two full-thickness wounds were made on the dorsal skin of mice using a 5 mm skin biopsy punch (Acu Punch, Acuderm, Ft. Lauderdale, FL). After placement of skin excisional wounds, 100 µl of the Invivofectamine 3.0 (Invitrogen, Carlsbad, CA) complex containing 1 nmol microRNA miR-21 mimic or non-targeting miRNA mimic (GE Healthcare Dharmacon, Lafayette, CO), anti-miR-10b LNA or negative control LNA (QIAGEN, Hilden, Germany) was directly injected into the surrounding dermis of the wound at four sites. Photographs of wounds were taken immediately after injury and then every day post-wounding until day 10 using a digital camera from a fixed distance with a ruler in field of view. Images were analyzed using AxioVision software (ZEISS, Oberkochen, Germany). The percent of wound closure was calculated as wound area/original wound area X 100%.

### MicroRNA profiling

Total RNA (including small RNA) was isolated from normal and wound tissue samples stored in RNALater using a miRNeasy Mini Kit (QIAGEN, Hilden, Germany). The microRNA profiling was then performed (in biological triplicates for each condition) using miR-Seq approach by LC Sciences, LLC (Houston, TX). In brief, small RNA libraries were generated using the Illumina Truseq Small RNA Preparation kit (San Diego, CA) according to manufacturer’s Sample Preparation Guide. The purified cDNA libraries were used for cluster generation on Illumina’s Cluster Station and then sequenced on the Illumina HiSeq platform. Raw sequencing reads (50 nt) were obtained using Illumina’s Sequencing Control Studio software version (v2.8) following real-time sequencing image analysis and base-calling by Illumina’s Real-Time Analysis (v1.8.70). The miR-Seq dataset has been submitted to Gene Expression Omnibus (GEO accession number: GSE121996).

### Bioinformatics analysis

A proprietary pipeline script, ACGT101-miR v4.2 g (LC Sciences, Houston, TX)^33–35^, was used for processing the miR-Seq data. In brief, the single end sequencing reads were cleaned with quality filter, adapter cutter, and length filter. The cleaned reads were mapped to miRBase (miRBase v21) using Bowtie v1.1.1^36^. Normalization of sequence counts in each sample was achieved by dividing sequence counts of individual samples with corresponding normalization factors which are the median values of the ratios between specific sample counts and geometric mean counts of all samples.

For differential analysis, t-test was used for between group comparison at baseline, one-way ANOVA was used for each time course data, and two-way ANOVA was used for between time course series comparison. The Benjamini-Hochberg adjusted P-values were computed for multiple hypothesis testing. Heatmaps were generated using Morpheus (https://software.broadinstitute.org/morpheus). Principal Component Analysis (PCA) and hierarchical clustering analysis were performed using Clustvis^37^. MicroRNA targeted pathway analysis were performed using DIANA-mirPath (v3.0)^38^, with microT-CDS^39,40^ as microRNA target prediction method (microT threshold = 0.8).

### Laser capture microdissection (LCM)

LCM was performed to isolate the epithelial cells from the wound edges using a LMD7000 Laser Microdissection system (Leica Microsystems, Wetzlar, Germany) as we previously described^41,42^.

### Real time PCR

MicroRNA real time PCR quantification was performed using TaqMan MicroRNA Assays (Applied Biosystems, Foster City, CA) according to manufacturer’s instruction. Data analysis was carried out using the 2^-delta delta Ct^ method^43^, where snRNA U6 was used as internal reference.

### Cell Culture and *in vitro* proliferation and migration assay

HaCaT (ATCC, Manassas, VA), a spontaneously transformed immortal keratinocyte cell line from skin was maintained in high glucose DMEM medium (Gibco) supplemented with 10% FBS, 100 units/ml penicillin, and 100 mg/ml streptomycin (Invitrogen), and TIGK (gift from Dr. Richard J. Lamont, University of Louisville), a telomerase immortalized epithelial cell line from oral mucosa (gingiva) was grown in DermaLife K medium (Lifeline Cell Technology) supplemented Life Factors (Lifeline Cell Technology), as previously described^27,44^. For functional analysis, miR-21 mimic and a control non-targeting miRNA mimic (GE Healthcare Dharmacon, Lafayette, CO), or anti-miR-10b LNA inhibitor and negative control LNA (QIAGEN, Hilden, Germany), were transfected into cells using DharmaFECT Transfection Reagent 1 as described previously^45–47^. Cell proliferation was measured using the MTT [3-(4,5-dimethylthiazol-2-yl)-2,5-diphenyl-2H-tetrazolium bromide] assay as described previously^26,48^. *In vitro* cell migration was measured using the Oris 96-well cell migration assay kit (Platypus Technologies, Fitchburg, WI) as described previously^45^. The cells that migrated into the detection zone were counted using ImageJ analysis software (version 1.421).

## Supplementary information

Supplementary Info
