# Supplementary material for: Differential microRNA profile underlies the divergent healing responses in skin and oral mucosal wounds
Source: Sci Rep. 2019 May 9;9:7160. doi: 10.1038/s41598-019-43682-w (PMC6509259; doi:10.1038/s41598-019-43682-w)
Supplement: Supplementary file 1 — Supplementary Info [file 41598_2019_43682_MOESM1_ESM.pdf]

Differential microRNA profile underlies the divergent healing responses in skin and oral mucosal wounds

Alyne Simões[1,2], Lin Chen[1], Zujian Chen[3], Yan Zhao[1], Shang Gao[4], Phillip T. Marucha[1,5], Yang Dai[4], Luisa A. DiPietro[1,6,*], Xiaofeng Zhou[1,3,6,7,*]

1. Center for Wound Healing & Tissue Regeneration, Department of Periodontics, College of Dentistry, University of Illinois at Chicago, Chicago, IL, USA.
2. Oral Biology Laboratory, Department of Biomaterials and Oral Biology, School of Dentistry, University of São Paulo, São Paulo, SP, Brazil.
3. Center for Molecular Biology of Oral Diseases, Department of Periodontics, College of Dentistry, University of Illinois at Chicago, Chicago, IL, USA.
4. Department of Bioengineering, College of Engineering, University of Illinois at Chicago, Chicago, IL, USA.
5. College of Dentistry, Oregon Health and Sciences University, Portland, OR, USA.
6. Graduate College, University of Illinois at Chicago, Chicago, IL, USA.
7. UIC Cancer Center, University of Illinois at Chicago, Chicago, IL, USA.

* Co-corresponding Authors:

X. Zhou, University of Illinois at Chicago, College of Dentistry, 801 S. Paulina Street, Chicago, IL 60612, USA. Email: xfzhou@uic.edu

L.A. DiPietro, University of Illinois at Chicago, College of Dentistry, 801 S. Paulina Street, Chicago, IL 60612, USA. Email: ldipiet@uic.edu

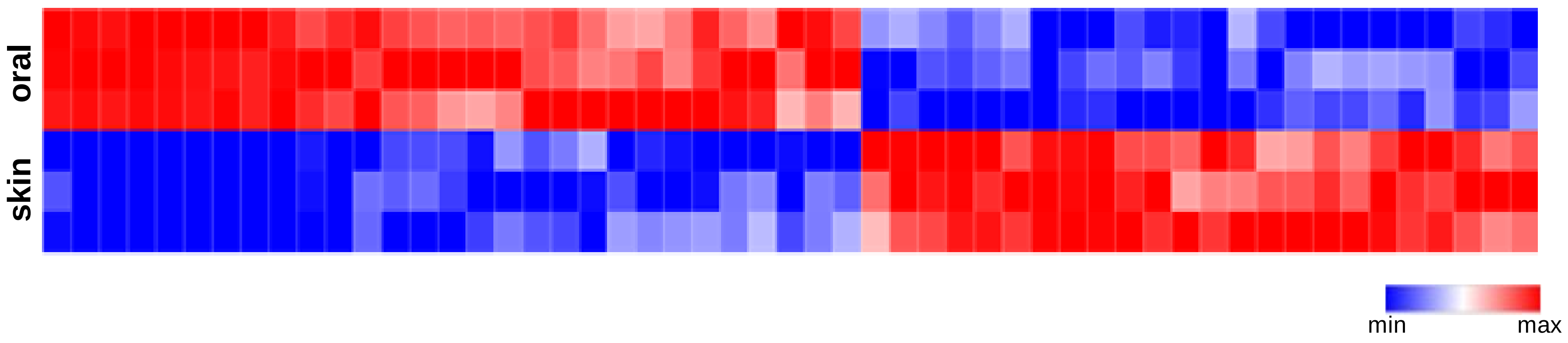

Supplementary Figure S1: Tissue specific microRNA signatures for skin and oral mucosa epithelium. MicroRNA profiling was performed on mouse skin and oral mucosal (palate) epithelium. Tissue specific microRNA signatures (presented as heatmap) for skin and oral mucosa epithelium were established by their differentially expression patterns (53 microRNAs, $p < 0.01$).

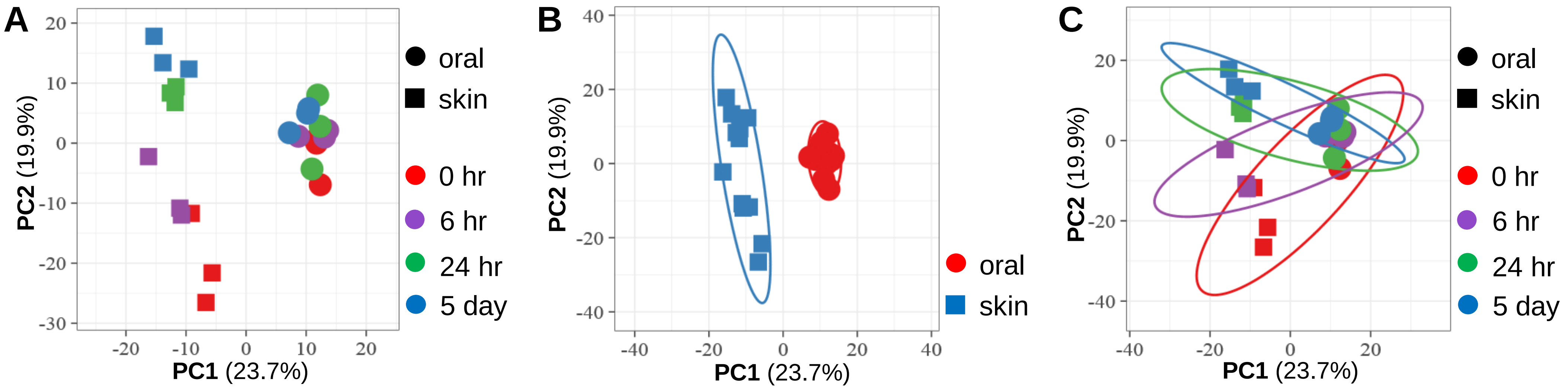

Supplementary Figure S2: Testing the classification potential of miRNome by unsupervised learning method to find grouping in samples set. Principal component analysis (PCA) was performed using the complete dataset (n=24), and PC1 and PC2 explain 23.7% and 19.9% of the variance, respectively (**A**). Prediction ellipses with probability of 0.95 were shown for tissue groups (**B**) and time group (**C**), indicate that PC1 can explain the variance associated with tissue specificity and PC2 appears to be associated with the variance related to wound healing time course.

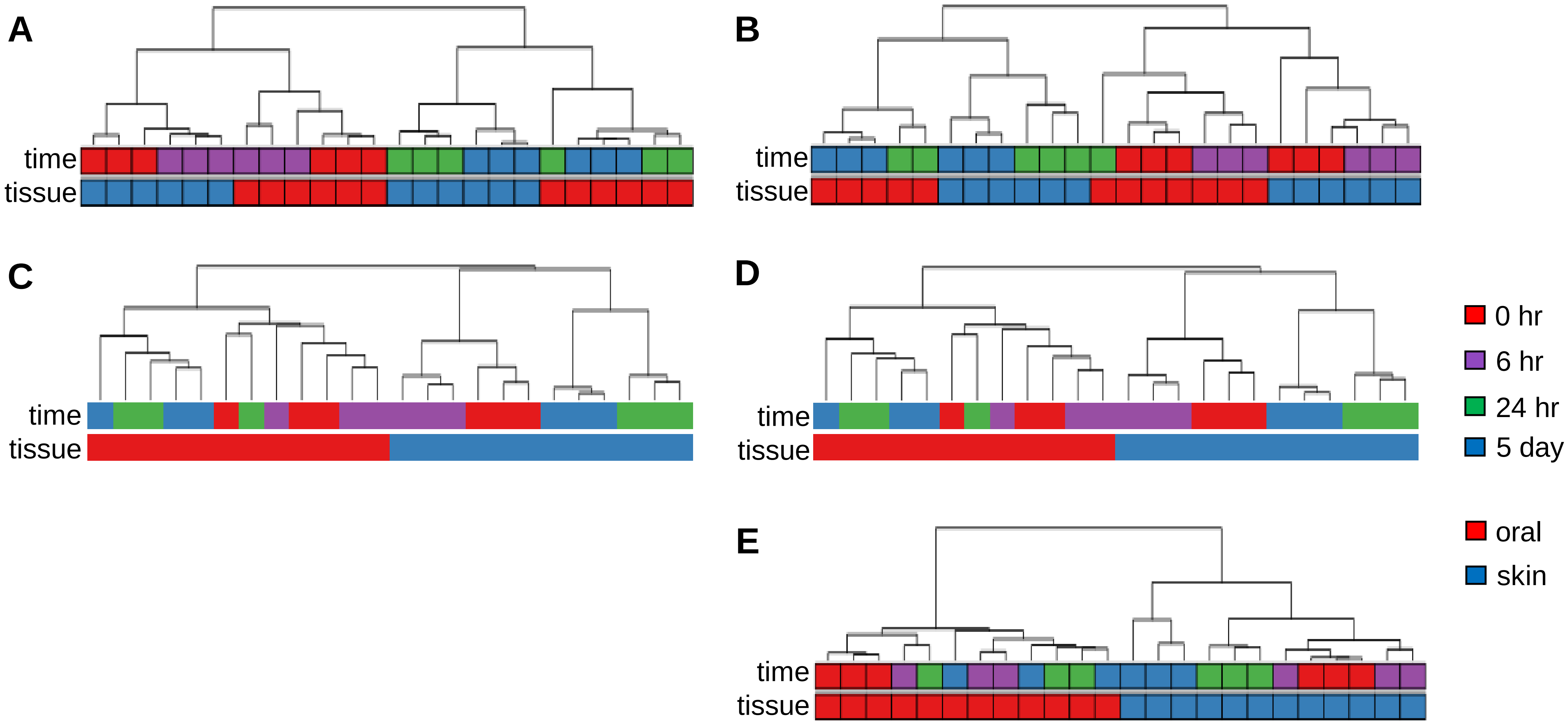

Supplementary Figure S3: Testing the classification potential of microRNA signature sets by unsupervised learning method to find grouping in samples set. Hierarchical clustering analysis was performed on the whole sample set (n=24), using (**A**) 18 concurrently differentially expressed microRNAs associated with both skin and oral wound healing, (**B**) 33 differentially expressed microRNAs associated with oral wound healing, (**C**) 200 differentially expressed microRNAs associated with skin wound healing, (**D**) the combined 215 differentially microRNAs in wound healing, (**E**) 53 baseline tissue specific differentially expressed microRNAs.

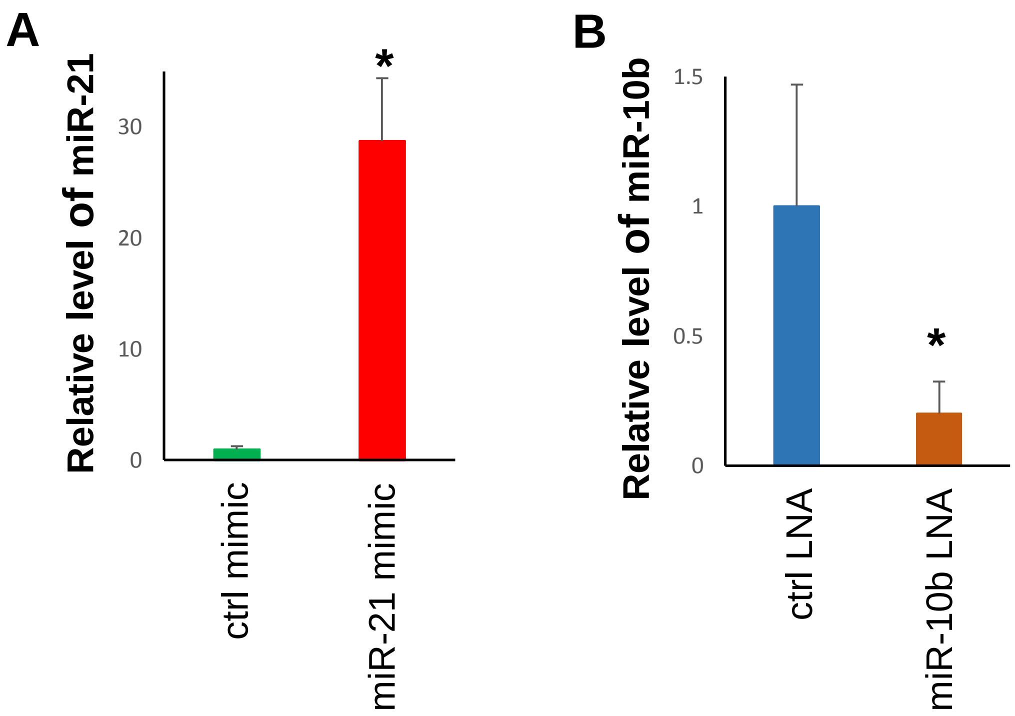

Supplementary Figure S4: Confirmation of the microRNA mimic mediated up-regulation of miR-21 and the LNA inhibitor mediated down-regulation of miR-10b in mouse skin wounds. (**A**) The miR-21 mimic or negative control mimic were delivered to the mouse skin wounds using an animal origin-free lipid nanoparticle (Invivofectamine 3.0 Reagent from Invitrogen) at the time of injury. The microRNA mimic mediated up-regulation of miR-21 was confirmed by TaqMan assay-based real time PCR quantification on day 1 wound samples. (**B**) The LNA inhibitor for miR-10b or negative control LNA were delivered to the mouse skin wounds using an animal origin-free lipid nanoparticle (Invivofectamine 3.0 Reagent from Invitrogen) at the time of injury. The LNA inhibitor mediated down-regulation of miR-10b was confirmed by TaqMan assay-based real time PCR quantification on wound tissue samples harvested at day 1 post-wounding (* $p < 0.05$).

Supplementary Table S1: Top 10 most abundant microRNAs in skin and oral mucosa during wound healing

would healing time course	Skin			oral mucosa		
	miR name	% of miRNome		miR name	% of miRNome	
0 hour (unwounded, baseline)	miR-10b	0.13453		miR-27b	0.096029	
	miR-10a	0.076385		miR-26a	0.063469	
	miR-26a	0.065607		miR-99a	0.059831	
	miR-27b	0.060006		miR-143	0.04909	
	miR-99a	0.040932		let-7c	0.045329	
	miR-143	0.031033		miR-125b	0.045091	
	miR-125b	0.030901		let-7f	0.034871	
	let-7c	0.030461		miR-199a	0.032343	
	let-7f	0.029307		miR-205	0.029648	
	miR-1a	0.026512		miR-203	0.029631	
	miR-21	0.010946	*	miR-21	0.013492	*
	miR-203	0.019338	*	miR-10b	0.028247	*
				miR-10a	0.000472	*
6 hours post wounding	miR-10b	0.116332		miR-27b	0.125659	
	miR-1a	0.073676		miR-99a	0.066628	
	miR-10a	0.065049		miR-26a	0.059918	
	miR-27b	0.060364		miR-143	0.057865	
	miR-99a	0.058471		let-7f	0.039192	
	miR-26a	0.052577		let-7c	0.037748	
	miR-143	0.039734		miR-125b	0.034433	
	let-7f	0.037163		miR-203	0.033448	
	miR-133a	0.027194		miR-199a	0.031841	
	miR-99b	0.022871		miR-21	0.024844	
	miR-21	0.020463	*			
24 hours post wounding	miR-21	0.064331		miR-27b	0.110127	
	miR-10b	0.052373		miR-99a	0.053833	
	miR-27b	0.050933		miR-26a	0.053736	
	miR-10a	0.049371		miR-21	0.046823	
	miR-99a	0.046014		let-7c	0.044605	
	let-7f	0.04268		miR-143	0.042485	
	miR-26a	0.040699		let-7f	0.036187	
	miR-146b	0.039564		miR-199a	0.028033	
	miR-143	0.03701		miR-203	0.027665	
	let-7c-5p	0.03682		miR-205	0.027606	
5 days post wounding	miR-21	0.129953		miR-27b	0.115615	
	miR-27b	0.060603		let-7c	0.051672	
	miR-10b	0.037804		miR-143	0.051497	

	let-7f	0.036286		miR-99a	0.051173
	miR-26a	0.036118		miR-26a	0.047261
	miR-143	0.033552		miR-21	0.040623
	miR-199a	0.033522		let-7f	0.038901
	let-7c	0.032773		miR-199a	0.031018
	miR-99a	0.030027		miR-203	0.030965
	miR-146b	0.030023		miR-125b	0.028131

note:

*: While not in top 10, these selected miRs were also presented for comparancing between tissues.

Supplementary Table S2: Baseline tissue-specific differentially expressed microRNAs between skin and oral mucosa epithelium

			Skin		Oral		
**miR name**	**p-value**	**adj P**	**Mean**	**StDev**	**Mean**	**StDev**	**miR_seq**
**miR-378a-3p**	1.29E-03	0.04304	15,167	183	6,013	400	ACTGGACTTGGAGTCAGAAGGC
**miR-31-5p**	1.85E-03	0.0523	437	140	9,980	739	AGGCAAGATGCTGGCATAGCT
**miR-10a-5p**	2.19E-03	0.05383	294,829	38,847	1,421	665	TACCCTGTAGATCCGAATTTGT
**miR-10b-5p**	2.52E-03	0.05931	519,259	82,583	85,095	24,872	TACCCTGTAGAACCGAATTTGT
**miR-133a-3p**	4.19E-03	0.0818	55,766	31,546	3,956	1,681	TTTGGTCCCCTTCAACCAGCTGT
**miR-146b-5p**	4.92E-03	0.08283	66,712	6,568	10,796	3,023	TGAGAACTGAATTCCATAGGCTGT
**miR-126a-5p**	6.54E-03	0.096	3,437	491	6,408	985	CATTATTACTTTTGGTACGCG
**miR-34c-5p**	7.34E-03	0.09894	122	43	10,229	8,453	AGGCAGTGTAGTTAGCTGATTGC
**miR-30a-5p**	8.14E-03	0.09918	47,463	4,893	29,008	1,047	TGTAAACATCCTCGACTGGAAGCT
**miR-96-5p**	8.19E-03	0.09918	11,975	1,738	6,919	917	TTTGGCACTAGCACATTTTTGCT
**miR-1a-3p**	8.30E-03	0.09918	102,330	27,364	9,517	5,590	TGGAATGTAAAGAAGTATGTAT
**miR-451a**	9.75E-03	0.10566	823	249	16,627	11,565	AAACCGTTACCATTACTGAGT
miR-470-5p	2.90E-05	0.00801	0	0	344	40	TTCTTGGACTGGCACTGGTGAGT
miR-465b-3p	4.23E-05	0.00801	0	0	21	2	GATCAGGGCCTTTCTAAGTAGA
miR-465c-5p	4.24E-05	0.00801	0	0	73	9	TATTTAGAATGGCGCTGATCTG
miR-10a-3p	7.10E-05	0.01005	51	8	0	0	CAAATTCGTATCTAGGGGAAT
miR-881-3p	1.35E-04	0.01134	0	0	54	11	AACTGTGTCTTTTCTGAATAGA
miR-465a-5p	1.47E-04	0.01134	0	0	106	27	TATTTAGAATGGCACTGATGTGA
miR-107-3p	1.58E-04	0.01134	842	50	442	23	AGCAGCATTGTACAGGGCTATC
miR-1247-3p	1.60E-04	0.01134	0	0	24	6	CGGGAACGTCGAGACTGGAGC
miR-880-3p	2.50E-04	0.01574	0	0	55	15	TACTCCATCCTCTCTGAGTAGA
miR-211-5p	3.99E-04	0.01965	423	87	42	10	TTCCCTTTGTCATCCTTTGCCT
miR-1247-5p	4.36E-04	0.01965	12	5	601	286	ACCCGTCCCGTTCGTCCCCGGA
miR-471-5p	4.46E-04	0.01965	0	0	22	9	TACGTAGTATAGTGCTTTTCAC
miR-351-5p	4.51E-04	0.01965	1,991	168	4,097	329	TCCCTGAGGAGCCCTTTGAGCCT
miR-132-3p	8.69E-04	0.0335	34	1	122	13	TAACAGTCTACAGCCATGGTCG
miR-214-3p	8.88E-04	0.0335	2,337	229	4,735	453	ACAGCAGGCACAGACAGGCAGT
miR-1983	1.20E-03	0.04229	30	6	111	17	CTCACCTGGAGCATGTTTTCT
miR-34c	1.79E-03	0.0523	0	0	141	116	AGGCAGTGTAGTTAGCTGATTGT
miR-338-3p	1.84E-03	0.0523	470	59	1,124	77	TCCAGCATCAGTGATTTTGTT
miR-872-3p	2.02E-03	0.05383	339	31	576	41	TGAACTATTGCAGTAGCCTCCT
miR-378b_R	2.10E-03	0.05383	3,066	241	1,296	186	ACTGGACTTGGAGTCAGAAGGCT
miR-150-5p	2.68E-03	0.06073	1,669	199	3,217	367	TCTCCCAACCCTTGTACCAGT
miR-193b-3p	3.54E-03	0.07707	3,812	315	2,245	249	AACTGGCCCACAAAGTCCCGCT
miR-1a-1-5p	3.97E-03	0.0818	47	26	3	2	ACATACTTCTTTATATGCCCATA
miR-467b-5p	4.21E-03	0.0818	5	3	86	50	TAAGTGCCTGCATGTATATGC
miR-218-5p	4.34E-03	0.0818	128	24	294	51	TTGTGCTTGATCTAACCATGT
miR-375-3p	4.67E-03	0.08283	277	43	1,377	450	TTTGTTCGTTCGGCTCGCGTGA
miR-133a-5p	4.81E-03	0.08283	272	3	25	7	AGCTGGTAAAATGGAACCAAAT
miR-676-5p	4.98E-03	0.08283	76	10	38	2	ACTCTACAACCTTAGGACTTGC
miR-672-5p	5.18E-03	0.0838	17	5	79	26	TGAGGTTGGTGTACTGTGTGTGA
miR-511-5p	5.43E-03	0.08531	19	3	4	1	CATGCCTTTTGCTCTGCACTCA
miR-128-3p	6.27E-03	0.09588	954	60	398	69	TCACAGTGAACCGGTCTCTTT
miR-196a-5p	6.61E-03	0.096	186	216	0	0	TAGGTAGTTTCATGTTGTTGG
miR-223-3p	6.86E-03	0.09705	706	134	289	24	TGTCAGTTTGTCAAATACCCCAT
miR-184-3p	7.25E-03	0.09894	41	5	22	3	TGGACGGAGAACTGATAAGGGT
miR-378d	7.63E-03	0.09918	1,332	134	368	96	ACTGGACTTGGAGTCAGAAGGT
miR-23a-5p	7.94E-03	0.09918	5	2	16	4	GGGGTTCCTGGGGATGGGATTT
miR-223-5p	8.56E-03	0.09918	105	15	19	6	CGTGTATTTGACAAGCTGAGTTGGACACT
miR-501-3p	8.59E-03	0.09918	3,835	392	1,609	327	AATGCACCCGGGCAAGGATTTGG
miR-669a-5p	8.81E-03	0.09972	5	2	28	3	AGTTGTGTGTGCATGTTCATGTCT

miR-874-3p	9.87E-03	0.10566	146	30	308	48	CTGCCCTGGCCCGAGGGACCGAC
miR-1198-5p	9.89E-03	0.10566	162	14	224	19	TATGTGTTCCTGGCTGGCTTGG

notes:

Bold miR name: differentially expressed miRs that have the reads greater than the average (5458) of the dataset.

adj P: Benjamini-Hochberg adjusted P value.

Supplementary Table S3: Differentially expressed microRNAs in oral mucosal wound healing

			0hr		6hr		24h		5dy		
**miRNA**	**p-value**	**adj P**	**Fold Chang**	**StDev**	**Fold Chang**	**StDev**	**Fold Chang**	**StDev**	**Fold Chang**	**StDev**	**miR_seq**
rno-miR-378a-3p	0.0008184	0.0867151	1	0.0664713	0.7328313	0.0347267	0.9548818	0.05151	1.0301608	0.09042	ACTGGACTTGGAGTCAGAAGGC
mmu-miR-148b-3p	0.0035625	0.1320058	1	0.0999046	1.3048705	0.4011814	2.4221855	0.9691142	3.2551242	0.6331512	TCAGTGCATCACAGAACTTTGT
mmu-miR-181a-5p	0.0044725	0.1320058	1	0.1137351	0.8214249	0.0947066	0.6598383	0.0646125	0.7023587	0.0548996	AACATTCAACGCTGTCGGTGAGT
mmu-miR-31-5p	0.0066523	0.1516625	1	0.0740672	0.8015539	0.2209285	0.9452544	0.0772447	1.4866995	0.1154021	AGGCAAGATGCTGGCATAGCT
mmu-miR-23b-3p	0.0071932	0.1516625	1	0.111629	0.7357408	0.0466384	1.0309673	0.1224344	0.9944933	0.0684041	ATCACATTGCCAGGGATTACC
mmu-miR-200c-3p	0.0092364	0.1632779	1	0.1557818	0.8651189	0.2146033	1.7654788	0.5850174	1.8783663	0.1905794	TAATACTGCCGGGTAATGATGGA
mmu-miR-195a-5p	0.0097908	0.1632779	1	0.1120782	0.9076189	0.0649711	0.8820817	0.1469965	0.6635547	0.0478765	TAGCAGCACAGAAATATTGGC
mmu-miR-3068-5p	0.0002268	0.0752639	1	0.257308	1.0663981	0.3900415	3.5321486	0.8150827	3.6866457	0.1352283	TTGGAGTTCATGCAAGTTCTAACC
mmu-miR-101c	0.0002632	0.0752639	1	0.1031532	3.6350439	0.4766979	1.6863383	0.5710504	1.4593673	0.2365202	TACAGTACTGTGATAACTGAT
mmu-miR-30a-3p	0.0006925	0.0867151	1	0.0882855	1.0545809	0.0647274	0.7847491	0.0160182	0.8296518	0.0335745	CTTTCAGTCGGATGTTTGCAGC
mmu-miR-17-5p	0.0007147	0.0867151	1	0.1584791	0.8475679	0.0483152	1.5117758	0.1628031	0.9948067	0.0465453	CAAAGTGCTTACAGTGCAGGTAG
mmu-miR-219a-5p	0.0009096	0.0867151	1	0.2515456	0.8655548	0.1150959	1.9265423	0.2487612	0.9787669	0.078554	TGATTGTCCAAACGCAATTCTCG
oan-miR-27b-3p	0.0013897	0.1075841	1	0.0529891	1.0604624	0.0815091	1.2212844	0.1148333	1.4813389	0.1309122	TTCACAGTGGCTAAGTTCTGCGT
mmu-miR-676-5p	0.001521	0.1075841	1	0.0587988	1.2536092	0.1310737	1.5343393	0.1647377	1.5822301	0.1767254	ACTCTACAACCTTAGGACTTGC
mmu-miR-187-5p	0.0020207	0.1075841	1	0.1332469	0.9633736	0.1775188	1.0897682	0.1082475	1.7067577	0.0787297	AGGCTACAACACAGGACCCGGGA
mmu-miR-136-5p	0.0020635	0.1075841	1	0.1548394	1.4044623	0.1419101	0.872338	0.052316	0.8988278	0.0852673	AGGACTCCATTTGTTTTGATGAT
mmu-miR-362-5p	0.0020689	0.1075841	1	0.3961423	0.8407961	0.4010792	3.2770347	1.3747622	4.2970719	0.8784176	AATCCTTGGAACCTAGGTGTGAAT
mmu-miR-434-3p	0.0023305	0.1110887	1	0.0890199	1.317616	0.0777199	0.7998577	0.1350153	0.9871484	0.0587686	TTTGAACCATCACTCGACTCCT
mmu-miR-20a-5p	0.002801	0.1232459	1	0.1382523	1.1142864	0.0608071	1.716838	0.3460544	1.4510558	0.0167519	TAAAGTGCTTATAGTGCAGGTAG
mmu-miR-425-3p	0.0035515	0.1320058	1	0.1509714	0.7956976	0.3416713	1.5800723	0.008423	1.9031004	0.134065	CATCGGGAATGTCGTGTCCGCC
mmu-miR-340-5p	0.0037138	0.1320058	1	0.0858626	2.052563	0.4916944	1.2777403	0.2398432	1.1007619	0.1596562	TTATAAAGCAATGAGACTGATT
mmu-miR-664-3p	0.0040783	0.1320058	1	0.0763262	0.9307747	0.0316889	0.7738323	0.146559	0.6382082	0.0549795	TATTCATTTACTCCCCAGCCT
mdo-miR-181a-5p	0.0045652	0.1320058	1	0.4062099	0.4520095	0.1849618	1.5972577	0.5706788	1.6012956	0.1129886	AACATTCAACGCTGTCGGTGAGTTTT
mmu-miR-700-5p	0.0046156	0.1320058	1	0.0989355	0.8064185	0.0942522	0.6528607	0.0928495	0.678155	0.0392187	TAAGGCTCCTTCCTGTGCTTGC
mmu-miR-7a-5p	0.0049464	0.13473	1	0.1152172	1.4026239	0.3423426	2.0379229	0.3815397	1.533827	0.1159376	TGGAAGACTAGTGATTTTGTTGTT
mmu-miR-193b-3p	0.0056089	0.1423618	1	0.1111418	0.5820916	0.0633109	0.6239657	0.1001622	0.5880681	0.1051606	AACTGGCCCACAAAGTCCCGCT
mmu-miR-141-5p	0.0057243	0.1423618	1	0.1374447	1.2939718	0.0981627	1.4636957	0.4518719	0.703996	0.0905252	CATCTTCCAGTGCAGTGTTGGA
chi-let-7b-5p	0.0060559	0.1443321	1	0.2975176	0.5674505	0.268898	0.7429631	0.2223544	0.2416335	0.0560828	CGTGGTGAGGTAGTAGGTTGTGTGGTT
mmu-miR-369-3p	0.0072728	0.1516625	1	0.3373636	1.8783307	0.4679148	0.8015508	0.0371642	1.3602938	0.1336945	AATAATACATGGTTGATCTTT
mmu-miR-223-3p	0.007424	0.1516625	1	0.0825289	2.6603169	0.8650528	3.7225306	2.4601821	1.0758219	0.174759	TGTCAGTTTGTCAAATACCCCAT
cgr-miR-2424	0.0086861	0.1632779	1	0.2404127	1.3771909	0.1189381	1.0305804	0.0909723	0.7900936	0.0090443	ACAGATCTTTGGTAATCTGATGGCT
mmu-miR-185-5p	0.0089071	0.1632779	1	0.1768065	0.8605288	0.093547	1.1757479	0.1890558	1.4661178	0.1582404	TGGAGAGAAAGGCAGTTCCTGA
mmu-miR-1981-5p	0.0089076	0.1632779	1	0.1363764	0.7991036	0.0798437	1.5570912	0.6208528	2.1169091	0.5442181	GTAAAGGCTGGGCTTAGACGTGG

notes:

adj P: Benjamini-Hochberg adjusted P value.

Supplementary Table S4: Differentially expressed microRNAs in skin wound healing

			0hr		6hr		24h		5dy		
**miRNA**	**p-value**	**BH adj p-valu**	**Fold Chang**	**StDev**	**Fold Chang**	**StDev**	**Fold Chang**	**StDev**	**Fold Chang**	**StDev**	**miR_seq**
mmu-miR-362-5p	4.65E-08	1.37615E-05	1	0.0837141	0.6413249	0.0891709	4.2812828	0.4271781	5.5743529	0.866634	AATCCTTGGAACCTAGGTGTGAAT
mmu-miR-378b	9.38E-07	0.000117099	1	0.5078893	101.84181	42.218462	68.367811	9.9917889	56.644035	8.8869892	CCTGGACTTGGAGTCAGAAGGC
mmu-miR-30a-5p	9.89E-07	0.000117099	1	0.1030876	1.2514485	0.111781	0.582879	0.0226006	0.453159	0.0342868	TGTAAACATCCTCGACTGGAAGCT
mmu-miR-21a-5p	1.90E-06	0.000187873	1	0.2539178	1.9940871	0.2800941	4.5685168	0.3442066	8.7049501	1.4660301	TAGCTTATCAGACTGATGTTGA
rno-miR-140-3p	2.82E-06	0.000238909	1	0.0324798	0.8162478	0.1297814	2.9362838	0.404611	1.6118772	0.037681	ACCACAGGGTAGAACCACGGACT
mmu-miR-132-3p	4.93E-06	0.000340665	1	0.0352686	1.5800502	0.2569972	3.4745052	0.9094494	7.8466167	1.5319354	TAACAGTCTACAGCCATGGTCG
mmu-miR-142a-5p	5.18E-06	0.000340665	1	0.0255513	1.2848074	0.2651946	4.1437431	0.4405762	2.3621902	0.3453455	CCCATAAAGTAGAAAGCACTAC
mmu-miR-574-5p	6.15E-06	0.000363805	1	0.1303411	1.0603561	0.0365244	1.7632596	0.1948124	2.3909489	0.1317313	TGAGTGTGTGTGTGTGAGTGT
mmu-miR-363-3p	8.53E-06	0.000459292	1	0.0952857	0.6652307	0.0891167	2.0053947	0.4497003	2.7139678	0.2054663	AATTGCACGGTATCCATCTGTAT
mmu-miR-148b-3p	1.21E-05	0.000596292	1	0.1225519	1.4401436	0.1791595	3.6437393	0.7899089	2.8325129	0.2177709	TCAGTGCATCACAGAACTTTGT
mmu-miR-370-3p	1.40E-05	0.000638568	1	0.0605291	1.3253023	0.1824486	1.0811611	0.4500462	7.6443345	1.0795915	GCCTGCTGGGGTGGAACCTGGT
mmu-miR-503-5p	1.76E-05	0.00074296	1	0.0705028	2.5582538	1.259011	13.009299	3.9726796	24.044095	4.5673285	TAGCAGCGGGAACAGTACTGCAG
mmu-miR-130b-5p	1.89E-05	0.000747309	1	0.3830448	0.7955666	0.0347566	4.3723402	0.7893309	2.7859502	0.2058123	ACTCTTTCCCTGTTGCACTACT
mmu-miR-223-5p	2.13E-05	0.000786602	1	0.1439168	1.0131108	0.2574665	4.9520961	0.3066759	1.9972864	0.5349822	CGTGTATTTGACAAGCTGAGTTGGACACT
mmu-miR-193b-3p	2.63E-05	0.000843812	1	0.0826412	0.7382197	0.0453673	0.528423	0.1066424	0.2426527	0.0544516	AACTGGCCCACAAAGTCCCGCT
mmu-miR-133a-5p	2.68E-05	0.000843812	1	0.0124961	1.6103408	0.6638912	0.6235684	0.0782252	0.2382276	0.0268378	AGCTGGTAAAATGGAACCAAAT
mmu-miR-409-3p	2.82E-05	0.000843812	1	0.1951633	1.643613	0.2753653	1.6411885	0.5257005	7.3129923	1.1643407	GAATGTTGCTCGGTGAACCCCT
mmu-miR-30e-3p	2.85E-05	0.000843812	1	0.1336761	1.0853182	0.0504217	0.7121563	0.0388258	0.4821073	0.0605488	CTTTCAGTCGGATGTTTACAGC
mmu-miR-540-3p	3.20E-05	0.000902329	1	0.303299	1.4690526	0.4930735	1.3405418	0.3674875	8.9795826	1.373148	AGGTCAGAGGTCGATCCTGGGC
mmu-miR-106b-3p	3.48E-05	0.00093549	1	0.1005209	1.0092353	0.1131692	3.1954698	0.7250982	2.2884583	0.2963492	CCGCACTGTGGGTACTTGCT
cgr-miR-139-5p	3.93E-05	0.00101248	1	0.1717709	1.5692417	0.2170089	6.2644141	1.1548753	1.8048574	0.5971037	TCTACAGTGCACGTGTCTCCAGT
mmu-miR-3068-5p	4.23E-05	0.001042265	1	0.1667673	0.6336216	0.051074	2.0222936	0.5268974	2.5359095	0.3824849	TTGGAGTTCATGCAAGTTCTAACC
mmu-miR-1198-5p	5.01E-05	0.001096217	1	0.0848818	1.2767261	0.0491863	2.4768726	0.5033243	1.9572109	0.151669	TATGTGTTCCTGGCTGGCTTGG
mmu-miR-7a-5p	5.29E-05	0.001096217	1	0.2993717	1.8734799	0.1347995	5.4050207	0.8134072	5.6690703	1.739785	TGGAAGACTAGTGATTTTGTTGTT
mmu-miR-1983	5.38E-05	0.001096217	1	0.1999005	1.1325902	0.4877763	3.0117198	0.5608882	8.178836	2.1482012	CTCACCTGGAGCATGTTTTCT
mmu-miR-31-5p	5.45E-05	0.001096217	1	0.3202481	0.6691327	0.4225293	0.8408116	0.3884696	11.688945	1.7979082	AGGCAAGATGCTGGCATAGCT
mmu-miR-195a-5p	5.56E-05	0.001096217	1	0.1482205	1.0863882	0.1409315	0.6123434	0.0445061	0.4327755	0.0546023	TAGCAGCACAGAAATATTGGC
mmu-miR-485-3p	5.93E-05	0.001133174	1	0.2459614	1.9433503	0.0360904	1.2241166	0.5878268	6.8873522	0.8276695	GTCATACACGGCTCTCCTCTCT
mmu-miR-223-3p	7.01E-05	0.001296402	1	0.1896275	3.4725465	1.0452788	21.881903	7.0927223	5.3417263	2.7982239	TGTCAGTTTGTCAAATACCCCAT
mmu-miR-667-3p	7.46E-05	0.001337691	1	0.1521384	0.8861784	0.191653	0.6646901	0.241756	3.5485176	0.349971	TGACACCTGCCACCCAGCCCAAG
mmu-miR-425-3p	8.08E-05	0.001402056	1	0.2348775	0.6035792	0.1815773	2.1053006	0.0998882	2.2176167	0.2181035	CATCGGGAATGTCGTGTCCGCC
mmu-miR-335-3p	8.29E-05	0.001402056	1	0.3701342	1.8152725	0.652019	0.9940331	0.1049006	6.5290917	1.3450705	TTTTTCATTATTGCTCCTGACC
mmu-miR-433-3p	8.93E-05	0.00146842	1	0.2100516	1.708403	0.338726	1.6380816	0.5173994	5.8043222	0.7944497	ATCATGATGGGCTCCTCGGTGT
mmu-miR-30a-3p	1.03E-04	0.001644349	1	0.183004	1.2628488	0.156489	0.6681405	0.1129742	0.3797533	0.064559	CTTTCAGTCGGATGTTTGCAGC
mmu-miR-34c-5p	1.23E-04	0.00191319	1	0.3544813	1.8903948	0.3133666	3.4416824	0.5934597	35.217775	29.988102	AGGCAGTGTAGTTAGCTGATTGC
mmu-miR-298-5p	1.35E-04	0.001986375	1	0.1104028	1.5461294	0.6010547	2.0791757	0.5955064	8.4553965	1.8720677	GGCAGAGGAGGGCTGTTCTTCCC
mmu-miR-379-3p	1.38E-04	0.001986375	1	0.2696918	1.3416434	0.3230751	0.8762274	0.213002	4.2139488	0.7683176	TATGTAACATGGTCCACTAAC
mmu-let-7g-5p	1.44E-04	0.002025249	1	0.0380169	1.3297929	0.0565121	1.8645238	0.2929749	1.1722114	0.0564105	TGAGGTAGTAGTTTGTACAGTT
mmu-miR-3535	1.57E-04	0.002155566	1	0.2646319	1.1974208	0.3209042	0.4218387	0.082361	0.2401472	0.0560957	TGGATATGATGACTGATTACCTGAGA

mmu-miR-96-5p	1.60E-04	0.002158314	1	0.1451501	1.059792	0.3840414	0.3436007	0.0457457	0.2286068	0.049049	TTTGGCACTAGCACATTTTTGCT
hsa-miR-411-3p	1.81E-04	0.002375894	1	0.3764971	1.3391691	0.4468603	0.8979431	0.2188992	6.635335	1.6070453	TATGTAACACGGTCCACTAAC
mmu-miR-134-5p	1.93E-04	0.002477828	1	0.5045897	1.8306631	0.2552175	1.6669484	0.4641584	7.7421117	0.9986563	TGTGACTGGTTGACCAGAGGGG
mmu-miR-503-3p	1.97E-04	0.002477828	1	0.1669201	1.3625549	0.1656011	1.3606887	0.1713954	2.8326096	0.5163766	GAGTATTGTTTCCACTGCCTGG
mmu-miR-351-3p	2.21E-04	0.002697113	1	0.5160102	3.1651568	0.5740431	4.2904132	0.3168941	12.748582	3.9546619	GGTCAAGAGGCGCCTGGGAAC
mmu-miR-30d-5p	2.31E-04	0.002697113	1	0.0801116	1.0830755	0.0613761	0.9045143	0.0719118	0.6733213	0.04992	TGTAAACATCCCCGACTGGAAGCT
mmu-miR-467b-5p	2.32E-04	0.002697113	1	0.5601718	2.8944241	0.3901845	8.115511	3.272596	5.5245781	0.405032	TAAGTGCCTGCATGTATATGC
mmu-miR-1a-3p	2.35E-04	0.002697113	1	0.2674081	2.9644116	1.6124386	0.7510039	0.0723657	0.3253727	0.0818349	TGGAATGTAAAGAAGTATGTAT
mmu-miR-323-3p	2.39E-04	0.002697113	1	0.5585449	4.129499	0.7608464	3.4139647	1.6093895	15.024682	4.2041709	CACATTACACGGTCGACCTCT
mmu-miR-361-3p	2.41E-04	0.002697113	1	0.0792432	0.688706	0.0080252	1.0993344	0.162064	1.2191758	0.0198303	CCCCCAGGTGTGATTCTGATTTGT
mmu-miR-541-5p	2.59E-04	0.002844113	1	0.1833458	1.3577702	0.1383864	1.0916668	0.415887	5.4283139	1.4219507	AAGGGATTCTGATGTTGGTCACACT
mmu-miR-26a-5p	2.85E-04	0.003068298	1	0.2251968	0.8548666	0.1153536	0.4822476	0.0365683	0.4036757	0.0567906	TTCAAGTAATCCAGGATAGGCT
mmu-miR-494-3p	3.16E-04	0.003343007	1	0.4294394	2.1115554	0.5560297	0.7715996	0.3377576	8.4825276	3.232242	TGAAACATACACGGGAAACCTCT
mmu-miR-376b-3p	3.30E-04	0.003408051	1	0.3440436	1.6609537	0.6776486	0.7467926	0.2669873	5.7122662	0.8513435	ATCATAGAGGAACATCCACTT
mmu-miR-186-5p	3.34E-04	0.003408051	1	0.0690767	1.1457398	0.1549692	0.587867	0.0332535	0.6642662	0.106961	CAAAGAATTCTCCTTTTGGGCT
mmu-miR-212-5p	3.59E-04	0.003562603	1	0.3830206	2.1547657	0.4243423	3.2183497	0.9333186	6.9008617	1.8257686	ACCTTGGCTCTAGACTGCTTACT
mmu-miR-15b-3p	3.61E-04	0.003562603	1	0.0305556	0.8181714	0.1699805	2.3387035	0.6628641	2.2079041	0.2324212	CGAATCATTATTTGCTGCTCT
rno-miR-25-3p	3.67E-04	0.003562603	1	0.1632616	1.0748019	0.095391	2.2901618	0.4756695	1.6257507	0.0638482	CATTGCACTTGTCTCGGTCTGA
mmu-miR-543-3p	3.80E-04	0.003566687	1	0.7012131	2.5617578	0.5445745	2.1204736	0.2965955	12.495648	2.3040777	AAACATTCGCGGTGCACTTCTT
mmu-miR-204-5p	3.86E-04	0.003566687	1	0.1804009	1.0925747	0.2258802	0.8357314	0.1992158	0.229482	0.09897	TTCCCTTTGTCATCCTATGCCT
mmu-miR-200a-3p	4.00E-04	0.003642225	1	0.1610251	1.1346017	0.0737134	1.0736307	0.0458806	0.4867077	0.1251561	TAACACTGTCTGGTAACGATGT
cfa-miR-194	4.25E-04	0.003812687	1	0.3220983	1.197963	0.4374698	5.4768064	1.0668365	3.079215	0.826779	TGTAACAGCAACTCCATGTGGAT
mmu-miR-34b-3p	4.67E-04	0.004117098	1	0.0983948	2.4936436	0.4709371	3.3565731	1.2588176	31.724868	31.777169	AATCACTAACTCCACTGCCATC
mmu-miR-450b-5p	4.73E-04	0.004117098	1	0.4271449	1.822944	0.3300301	4.0121439	1.1600193	4.784724	1.1127844	TTTTGCAGTATGTTCCTGAATAC
mmu-mir-3470a-p3	5.06E-04	0.004255833	1	0.3114472	5.1495352	2.9759897	8.2700526	1.2078015	2.1132654	0.5770552	TGCCTCTGCCTCCTGAGTGC
mmu-miR-679-5p	5.06E-04	0.004255833	1	0.6020162	1.2697043	0.1811856	0.4040892	0.1382002	3.96496	1.2275843	TTGGACTGTGAGGTGACTCTTGGT
cgr-miR-139-3p	5.10E-04	0.004255833	1	0.6151021	1.3568197	0.1397321	7.1855223	1.3224754	3.8051502	1.2796347	TGGAGACGCGGCCCTGTTGGAGT
mmu-miR-125a-3p	5.21E-04	0.004265844	1	0.2462619	1.5251663	0.1751887	2.4627273	0.4619914	2.3824232	0.061039	ACAGGTGAGGTTCTTGGGAGC
hsa-mir-4450-p3	5.26E-04	0.004265844	1	0	423.0273	730.97273	30564.027	5904.1359	20063.524	6812.6168	CCCCTGGTCCCGTGG
mmu-miR-382-5p	5.34E-04	0.004274067	1	0.2798859	1.7164581	0.6450547	2.1983785	0.5719527	5.3355518	0.9304146	GAAGTTGTTCGTGGTGGATTCG
mmu-miR-152-5p	5.42E-04	0.004274247	1	0.0518277	0.7466772	0.0321612	0.7031543	0.1416423	1.5704891	0.2917459	TAGGTTCTGTGATACACTCCGACT
mmu-miR-877-5p	6.10E-04	0.004692327	1	0.3079597	1.4961093	0.0473247	2.0203964	0.3501595	2.5746913	0.1153542	GTAGAGGAGATGGCGCAGGGGACA
mmu-miR-130b-3p	6.27E-04	0.004755625	1	0.5063375	0.3902219	0.1459471	3.0441247	0.9317452	2.8090063	0.99998	CAGTGCAATGATGAAAGGGCAT
mmu-miR-125b-5p	6.75E-04	0.005008493	1	0.2615703	0.602216	0.1009059	0.3162235	0.0351576	0.4942301	0.0759235	TCCCTGAGACCCTAACTTGTGA
mmu-miR-145a-3p	6.77E-04	0.005008493	1	0.0664913	1.3256934	0.1505205	0.6756536	0.0903052	0.6451689	0.1304375	GGATTCCTGGAAATACTGTTCT
mmu-miR-1193-3p	7.06E-04	0.005131305	1	0.2535784	1.808854	0.6342052	0.6718077	0.2675109	3.7330045	0.8049799	TAGGTCACCCGTTTTACTATC
mmu-miR-181c-5p	7.11E-04	0.005131305	1	0.171084	0.902911	0.1426848	0.5856032	0.1623561	0.3183347	0.0794569	AACATTCAACCTGTCGGTGAGT
mmu-miR-669c-5p	7.53E-04	0.005368925	1	0.3905979	0.8600733	0.229576	3.8504176	1.1115969	2.461776	0.3822116	ATAGTTGTGTGTGGATGTGTGT
mmu-miR-21a-3p	7.62E-04	0.005369281	1	0.9340874	5.2760077	2.7618646	15.400221	7.0625357	20.108678	4.3897202	CAACAGCAGTCGATGGGCTGT
hsa-miR-320b	7.95E-04	0.005511112	1	0.2189228	1.1937658	0.1912847	1.4762131	0.3631229	0.4490728	0.1131755	AAAAGCTGGGTTGAGAGGGT
mmu-miR-365-3p	8.01E-04	0.005511112	1	0.30881	0.5384289	0.0563338	0.3219968	0.0683889	0.309191	0.062356	TAATGCCCCTAAAAATCCTTAT
mmu-miR-361-5p	8.31E-04	0.005651865	1	0.2585363	1.214897	0.108786	2.5187528	0.3208922	1.7109224	0.2354898	TTATCAGAATCTCCAGGGGTAC
mmu-miR-1a-1-5p	8.64E-04	0.005810023	1	0.5504198	1.6179643	0.6028675	0.7709123	0.4339074	0.1381317	0.0368482	ACATACTTCTTTATATGCCCATA

mdo-miR-22-3p	9.34E-04	0.006215807	1	0.2597698	3.4874247	0.4413912	2.1055126	0.1832964	2.034302	0.6535229	CAGCTGCCAGTTGAAGAACTGC
ptr-miR-203	9.53E-04	0.006216654	1	0.4441403	0.570919	0.0756663	0.3749706	0.0370697	0.2519709	0.0313367	TGAAATGTTTAGGACCACTAGAAT
ptr-let-7i	9.56E-04	0.006216654	1	0.089807	1.0789105	0.0827996	1.5217702	0.2550633	1.624975	0.0551918	TGAGGTAGTAGTTTGTGCTGTT
mmu-miR-30c-5p	9.80E-04	0.006306472	1	0.1339291	0.9809341	0.0244722	0.7775185	0.0205516	0.6348351	0.078099	TGTAAACATCCTACACTCTCAGCT
mmu-miR-99a-5p	9.96E-04	0.006316442	1	0.0842003	1.5237995	0.1828551	0.8738889	0.2086207	0.5379115	0.1481965	AACCCGTAGATCCGATCTTGT
mmu-miR-532-5p	1.00E-03	0.006316442	1	0.2361885	1.1308605	0.2618443	2.3098308	0.367316	2.316798	0.4282187	CATGCCTTGAGTGTAGGACCGT
mmu-miR-203-3p	1.02E-03	0.006378673	1	0.0320972	0.7815985	0.0756983	0.6285213	0.0475543	0.5676929	0.1051031	TGAAATGTTTAGGACCACTAGT
mmu-miR-369-5p	1.06E-03	0.006515823	1	0.1892327	1.906397	0.3355055	1.2184863	0.2815081	2.689006	0.4215147	AGATCGACCGTGTTATATTCGC
mmu-mir-3470b-p5	1.11E-03	0.006661251	1	0.0282929	1.07393	0.0937758	1.9358043	0.4643978	0.7823614	0.1765447	TGTAGCCCTGGCTGTCC
mmu-miR-133a-3p	1.11E-03	0.006661251	1	0.5656919	2.0078005	1.0507723	0.6972099	0.3142804	0.1621058	0.0153855	TTTGGTCCCCTTCAACCAGCTGT
mmu-miR-872-3p	1.14E-03	0.006738455	1	0.0921454	0.7905882	0.0106498	0.7117445	0.09328	1.1642097	0.1323534	TGAACTATTGCAGTAGCCTCCT
hsa-miR-4448	1.17E-03	0.006738455	1	0.7641477	1.5148669	1.040295	13.416246	5.3000464	4.1536743	0.8637108	GGCTCGTTGGTCTAGGGG
mmu-miR-10b-5p	1.17E-03	0.006738455	1	0.1590404	0.9224281	0.4031696	0.302635	0.0911346	0.2060538	0.0682445	TACCCTGTAGAACCGAATTTGT
mmu-miR-708-3p	1.17E-03	0.006738455	1	0.2572063	1.2745179	0.1359119	0.7035791	0.1237406	0.5078613	0.0593653	CAACTAGACTGTGAGCTTCTAGT
mmu-miR-125b-1-3p	1.19E-03	0.006757886	1	0.3020723	2.709657	0.0590845	1.7070669	0.2485054	1.6080386	0.1214718	ACGGGTTAGGCTCTTGGGAGCT
mmu-miR-5099	1.20E-03	0.006757886	1	0.1587364	1.2776844	0.336601	3.3656113	0.9662659	1.6002579	0.1353409	TCTTAGATCGATGTGGTGCTC
rno-miR-214-3p	1.23E-03	0.006857908	1	0.0981375	0.8395052	0.2117344	0.8169993	0.1111326	1.8510233	0.2991751	ACAGCAGGCACAGACAGGCAGT
mdo-miR-26-5pC	1.26E-03	0.006997458	1	0.316483	0.7956475	0.0435024	0.3702769	0.0857739	0.4072611	0.0795074	TTCAAGTAACCCAGGATAGGCT
mmu-miR-615-3p	1.30E-03	0.007111274	1	0.1234199	1.0080731	0.2256506	0.8355923	0.1584079	2.4102891	0.5818651	TCCGAGCCTGGGTCTCCCTCT
mmu-miR-340-3p	1.31E-03	0.007111274	1	0.223756	1.1499104	0.1054169	2.0666786	0.2216629	1.3222915	0.1005315	TCCGTCTCAGTTACTTTATAGC
mmu-miR-212-3p	1.34E-03	0.007211834	1	0.650503	1.7846136	0.7098364	5.9406308	2.5047141	7.6220823	1.8044612	TAACAGTCTCCAGTCACGGCC
mmu-miR-192-5p	1.39E-03	0.007418295	1	0.1520014	1.2029768	0.1076329	1.3479674	0.0514551	0.7931981	0.0929728	CTGACCTATGAATTGACAGCC
mmu-miR-337-5p	1.40E-03	0.007418295	1	0.1743153	1.8870253	0.6741124	1.1635705	0.1782623	3.2104453	0.5461503	CGGCGTCATGCAGGAGTTGATT
mmu-miR-673-5p	1.45E-03	0.007570487	1	0.3511423	1.2538674	0.3364797	0.5105595	0.1806137	2.4970974	0.3003384	CTCACAGCTCTGGTCCTTGGAGC
mmu-miR-146b-3p	1.48E-03	0.007673969	1	0.2384586	1.8645249	0.7113588	3.6254825	0.0704519	2.298739	0.3943435	GCCCTAGGGACTCAGTTCTGGT
mmu-miR-299b-3p	1.58E-03	0.008119127	1	0.5459631	1.8728109	0.4820067	1.1168657	0.2777005	4.8652691	1.3935738	GTATGTGGGACGGTAAACCGCT
mmu-miR-329-5p	1.63E-03	0.008214548	1	0.2896125	1.6732116	0.198918	0.8809755	0.2324953	2.4263452	0.4331832	AGAGGTTTTCTGGGTCTCTGTTT
mmu-miR-423-3p	1.63E-03	0.008214548	1	0.1233289	0.8636987	0.0333471	1.3855005	0.264183	1.4785747	0.0413327	AGCTCGGTCTGAGGCCCCTCAGT
mmu-miR-1195	1.64E-03	0.008214548	1	0.3114472	2.1630132	0.8272173	6.9081157	1.4070997	1.6468567	0.8384273	TGAGTTCGAGGCCAGCCTGGTC
mdo-miR-26-5p	1.67E-03	0.008284584	1	0.4683106	0.5418045	0.1753635	0.2301134	0.0138412	0.229103	0.0290966	CGTGGTTCAAGTAATCCAGGATAGGCT
mmu-miR-199a-5p	1.72E-03	0.008486035	1	0.0562269	0.7437197	0.1400344	0.4944118	0.1129725	1.2556766	0.2676846	CCCAGTGTTCAGACTACCTGTTC
mmu-miR-200c-3p	1.79E-03	0.008741844	1	0.2116411	0.5786912	0.1911222	1.2939722	0.2034201	1.6404653	0.1749315	TAATACTGCCGGGTAATGATGGA
mmu-miR-329-3p	1.82E-03	0.00874745	1	0.4003871	1.3164219	0.4512617	0.7721013	0.0459532	2.7046698	0.2062814	AACACACCCAGCTAACCTTTTT
mmu-miR-3068-3p	1.83E-03	0.00874745	1	0.0853513	1.3436764	0.1303298	0.8816311	0.1147326	0.7104505	0.1218952	GGTGAATTGCAGTACTCCAAC
mmu-miR-3473d	1.83E-03	0.00874745	1	0.0983948	0.6864788	0.4096885	1.5027072	0.221669	3.4271248	0.5691545	CACTGAGCCACTTTCCAGCCCT
mmu-miR-340-5p	1.90E-03	0.008992678	1	0.4536075	2.6121444	1.098281	6.9295512	2.1219381	3.4206165	1.0923573	TTATAAAGCAATGAGACTGATT
mmu-miR-125a-5p	1.93E-03	0.00905272	1	0.3378152	0.5212082	0.1040373	0.2903637	0.0310685	0.4707845	0.0916847	TCCCTGAGACCCTTTAACCTGT
mmu-miR-328-3p	1.94E-03	0.00905272	1	0.1269239	1.2531525	0.108406	1.073184	0.156667	0.7256735	0.0490708	CTGGCCCTCTCTGCCCTTCCGTT
mmu-miR-181d-5p	1.96E-03	0.00905272	1	0.1154041	0.8370233	0.1188889	0.7635974	0.0925107	0.531843	0.0667381	AACATTCATTGTTGTCGGTGGGT
mmu-miR-98-3p	1.99E-03	0.009087899	1	0.2002938	0.9050202	0.1561054	0.9469613	0.2892732	2.4031906	0.3844809	CTATACAACTTACTACTTTCCT
mmu-miR-335-5p	2.00E-03	0.009087899	1	0.1500362	1.5836286	0.3175577	0.3961481	0.2564381	1.6974553	0.4220364	TCAAGAGCAATAACGAAAAATGT
mmu-miR-221-3p	2.05E-03	0.009198708	1	0.0451682	0.9441157	0.0433964	1.4548521	0.2312642	1.3346873	0.1339009	AGCTACATTGTCTGCTGGGTTTC
mmu-miR-132-5p	2.07E-03	0.009198708	1	0.7180719	4.5208297	2.2352513	8.8163452	2.5033658	15.456815	2.1802049	ACCGTGGCTTTCGATTGTTACT

mmu-miR-30c-2-3p	2.08E-03	0.009198708	1	0.1992231	0.9653194	0.0988994	0.6472745	0.1060463	0.453801	0.0974637	CTGGGAGAAGGCTGTTTACTCT
mmu-miR-193b-5p	2.08E-03	0.009198708	1	0.0636888	0.9340729	0.098463	0.5763472	0.1918744	0.4791185	0.0592132	CGGGGTTTTGAGGGCGAGATGA
cgr-miR-214-5p	2.11E-03	0.00923999	1	0.2179683	0.8060234	0.100771	0.7994889	0.1596177	1.6202021	0.0989944	TGCCTGTCTACACTTGCTGTGC
mmu-miR-152-3p	2.18E-03	0.009485439	1	0.2107579	1.331513	0.1110373	1.2382011	0.116605	1.7990415	0.0460345	TCAGTGCATGACAGAACTTGG
mmu-miR-10a-5p	2.23E-03	0.009632311	1	0.1317609	0.9084143	0.3592673	0.5024623	0.1548674	0.2383207	0.0880976	TACCCTGTAGATCCGAATTTGT
mmu-miR-10a-3p	2.25E-03	0.009632311	1	0.1532046	0.9745423	0.1714622	1.9002149	0.2383496	1.2308395	0.1886834	CAAATTCGTATCTAGGGGAAT
mmu-miR-674-5p	2.27E-03	0.009649775	1	0.1573378	0.7600588	0.1507554	1.231239	0.0935557	1.5412692	0.2082108	GCACTGAGATGGGAGTGGTGTA
mmu-miR-434-5p	2.38E-03	0.010064861	1	0.3704331	1.8505936	0.1136602	0.622515	0.2264647	1.9643819	0.349352	GCTCGACTCATGGTTTGAACCA
chi-miR-99b-3p	2.46E-03	0.010323635	1	0.1695248	0.9689348	0.1332949	1.5792321	0.1267033	2.1772204	0.6362701	CAAGCTCGTGTCTGTGGGTCCGT
mmu-miR-351-5p	2.51E-03	0.010465544	1	0.0844893	1.4586638	0.3793304	0.9875856	0.2087979	2.4672966	0.6070181	TCCCTGAGGAGCCCTTTGAGCCT
mmu-miR-379-5p	2.58E-03	0.010688997	1	0.6197519	2.6005561	0.6625438	2.7930513	0.5480395	4.5700396	0.6675896	TGGTAGACTATGGAACGTAGG
hsa-miR-4454	2.71E-03	0.011135146	1	0.311402	1.1938938	0.1763517	2.3062566	0.152184	1.8604314	0.3211017	TCGGATCCGAGTCACGGCACCA
mmu-miR-664-3p	2.86E-03	0.011691663	1	0.4108561	1.3226349	0.1018787	0.6945605	0.123068	0.3892615	0.0571229	TATTCATTTACTCCCCAGCCT
mmu-miR-665-3p	2.89E-03	0.011721564	1	0.6152815	1.8785085	1.0785012	1.2135596	0.7321664	7.6085242	0.9796893	ACCAGGAGGCTGAGGTCCCTTA
mmu-miR-148b-5p	2.94E-03	0.011779138	1	0.2371952	1.5091068	0.1941134	1.6384198	0.4292438	2.5139179	0.2235382	GAAGTTCTGTTATACACTCAGGC
mmu-miR-107-3p	2.94E-03	0.011779138	1	0.0588604	0.7637262	0.043722	0.7815476	0.1566574	0.5728624	0.0601727	AGCAGCATTGTACAGGGCTATC
mmu-miR-574-3p	3.08E-03	0.012233153	1	0.159976	0.7226952	0.056914	0.6026445	0.0536147	0.7100194	0.0608214	CACGCTCATGCACACACCCACA
mmu-miR-20a-5p	3.18E-03	0.012458717	1	0.2842205	1.3300627	0.2187253	2.704159	0.4683716	2.3073174	0.6753191	TAAAGTGCTTATAGTGCAGGTAG
rno-miR-181a-2-3p	3.20E-03	0.012458717	1	0.2653292	1.5079592	0.560857	1.0365957	0.1916379	0.4258602	0.1266605	ACCACCGACCGTTGACTGTACC
mmu-miR-24-2-5p	3.29E-03	0.012747155	1	0.0415376	0.7326629	0.0577254	0.5196467	0.1204124	0.7367385	0.0788055	GTGCCTACTGAGCTGAAACAGT
mmu-miR-210-3p	3.34E-03	0.012844095	1	0.3060559	0.7264773	0.0703653	1.4910294	0.5197572	2.3399945	0.5231143	CTGTGCGTGTGACAGCGGCTGA
mmu-miR-484	3.53E-03	0.013400543	1	0.1607864	0.6242116	0.0959694	0.5689294	0.1027721	0.9602006	0.0738081	TCAGGCTCAGTCCCCTCCCGAT
mmu-miR-221-5p	3.53E-03	0.013400543	1	0.123451	1.3830767	0.4084483	0.7507778	0.1707078	0.5391125	0.0653802	ACCTGGCATACAATGTAGATTTCTGT
rno-miR-378a-5p	3.68E-03	0.013885205	1	0.1162085	1.2795807	0.4581227	0.7671961	0.1018661	0.4619925	0.1063637	CTCCTGACTCCAGGTCCTGTGT
mmu-miR-15b-5p	3.92E-03	0.01454485	1	0.254236	0.9961632	0.1559138	1.9864855	0.2866396	1.6486097	0.2356136	TAGCAGCACATCATGGTTTACA
mmu-mir-3102-p3	3.93E-03	0.01454485	1	0.1217697	1.1231961	0.0398356	1.2453359	0.1695152	1.7391794	0.2926564	GAGCACCCCATTGGCTACCCACA
rno-miR-25-5p	4.02E-03	0.014777333	1	0.2642865	0.5820014	0.1125325	1.3843085	0.3165579	1.3872368	0.2148243	AGGCGGAGACTTGGGCAATTGCT
rno-miR-874-3p	4.04E-03	0.014777333	1	0.203448	1.6711652	0.1747096	0.9866017	0.1144491	1.2519861	0.0609583	CTGCCCTGGCCCGAGGGACCGAC
mmu-miR-1839-5p	4.17E-03	0.015161566	1	0.0349554	1.2111516	0.0284152	1.3177189	0.1793922	0.9655972	0.063744	AAGGTAGATAGAACAGGTCTTGT
mmu-miR-3970	4.21E-03	0.015189612	1	0.0293925	1.6609021	0.6928908	2.5055393	0.1968514	1.2403072	0.1757495	TGAGGTAGTAGTTTGTGCTT
mmu-miR-211-5p	4.24E-03	0.015211967	1	0.2066138	0.5482685	0.2593193	0.4384779	0.0998019	0.2386098	0.0661386	TTCCCTTTGTCATCCTTTGCCT
mmu-miR-495-3p	4.32E-03	0.015413002	1	0.408068	1.5209092	0.4491724	0.8112002	0.2079628	3.2814585	0.9815277	AAACAAACATGGTGCACTTCTT
mmu-miR-1247-5p	4.37E-03	0.015475064	1	0.3893578	0.8500563	0.6325552	1.5219218	0.494682	8.504674	2.8816599	ACCCGTCCCGTTCGTCCCCGGA
mmu-miR-300-3p	4.56E-03	0.016053992	1	0.3589051	1.9686281	0.7736451	1.2421537	0.5566182	3.8856108	0.6304635	TATGCAAGGGCAAGCTCTCT
mmu-miR-28c	4.62E-03	0.016168779	1	0.038418	2.5820541	0.5936916	2.346044	0.6593746	2.1849821	0.7020198	AAGGAGCTCACAGTCTATTGAA
mmu-miR-99a-3p	4.67E-03	0.016241055	1	0.3122041	0.5129538	0.0365709	0.34673	0.0542963	0.4371538	0.1392292	CAAGCTCGTTTCTATGGGTCTGT
mmu-miR-23b-3p	4.69E-03	0.016241055	1	0.1794263	0.71159	0.0856286	0.6373715	0.1060278	0.4965126	0.0713242	ATCACATTGCCAGGGATTACC
mmu-miR-676-5p	4.75E-03	0.016241143	1	0.1366818	0.784872	0.1113213	0.6072587	0.0551504	0.6484576	0.0842404	ACTCTACAACCTTAGGACTTGC
mmu-miR-224-5p	4.75E-03	0.016241143	1	0.1463129	1.2226207	0.2474027	1.0545799	0.3656132	2.3652663	0.1931441	TAAGTCACTAGTGGTTCCGTTT
mmu-miR-712-5p	4.87E-03	0.016575598	1	0.2597698	0.8459174	0.5202588	2.1886211	0.4080564	4.0927353	0.7872991	CTCCTTCACCCGGGCGGTACCCGC
mmu-miR-382-3p	4.95E-03	0.01673073	1	0.1506408	1.7382395	0.7143872	1.2238049	0.5702819	3.5159739	0.6977001	AATCATTCACGGACAACACTTT
mmu-miR-222-3p	4.99E-03	0.016771314	1	0.1176075	1.1978463	0.2587777	2.0301863	0.5529001	1.8339041	0.1542446	AGCTACATCTGGCTACTGGGTCTCT
hsa-miR-7977	5.20E-03	0.017391405	1	0.3892841	0.7436631	0.5078532	3.682947	0.9320637	6.1648424	4.1107003	CCCGGCCAACGCACCA

mmu-miR-582-3p	5.29E-03	0.017519612	1	0.290331	1.0502508	0.1731728	2.1214745	0.0970939	1.7353721	0.4044018	AACCTGTTGAACAACTGAACCC
mmu-miR-342-3p	5.30E-03	0.017519612	1	0.1179536	0.9414646	0.0667833	0.8569387	0.2121842	1.5077798	0.1103269	TCTCACACAGAAATCGCACCCGT
mmu-miR-496a-3p	5.40E-03	0.017755774	1	0.4757403	2.0560373	0.5377885	1.0840162	0.4024521	3.6266225	0.7492749	AGTATTACATGGCCAATCTCC
mmu-miR-409-5p	5.61E-03	0.018344963	1	0.3589175	2.4981159	0.2016965	0.7784754	0.2953417	1.6410004	0.3760255	AGGTTACCCGAGCAACTTTGCAT
mmu-miR-194-5p	5.67E-03	0.018413039	1	0.2302868	1.386207	0.2040148	2.9052787	0.8708733	1.2588619	0.3962527	TGTAACAGCAACTCCATGTGGA
mmu-miR-6538	5.70E-03	0.018413039	1	0.2999702	1.9440028	1.1436939	7.041941	0.7296326	1.6147006	1.2666139	CGCGGGCTCCGGGGC
bta-miR-378	5.72E-03	0.018413039	1	0.1181157	2.224835	0.4944649	1.491496	0.2974698	1.4148635	0.2538082	CCTGGACTTGGAGTCAGAAGGCT
mmu-miR-17-5p	5.92E-03	0.018934664	1	0.3303307	1.225979	0.0878724	2.4548547	0.4177761	2.1884502	0.6963248	CAAAGTGCTTACAGTGCAGGTAG
mmu-miR-542-5p	6.09E-03	0.019328376	1	0.2671163	0.9438729	0.2503843	2.0347155	0.7322074	3.6774023	1.8229044	CTCGGGGATCATCATGTCACGA
mmu-miR-144-3p	6.11E-03	0.019328376	1	1.015959	15.691404	5.7180296	14.157613	5.8400912	9.5186871	7.2860747	TACAGTATAGATGATGTAC
mmu-miR-5126	6.17E-03	0.019437069	1	0.537093	1.3774477	1.2077884	5.6867813	2.6253156	11.344691	6.8201904	GCGCGGGCGGGGCCGGGGG
mmu-miR-450a-5p	6.53E-03	0.020443501	1	0.1633147	1.3582107	0.3034625	1.7743298	0.5885427	2.4361341	0.2770026	TTTTGCGATGTGTTCCTAATAT
chi-miR-326-5p	6.73E-03	0.020875338	1	0.135016	0.8610848	0.3711388	3.5847342	1.1754849	2.472339	1.33005	CCTCATCTGTCTGTTGGGCT
mmu-miR-145a-5p	6.74E-03	0.020875338	1	0.3201111	0.5908406	0.1324421	0.4066126	0.0381804	0.4020431	0.0867146	GTCCAGTTTTCCCAGGAATCCCT
mmu-miR-26a-2-3p	7.08E-03	0.021675393	1	0.281269	0.8414142	0.1863195	0.4389714	0.0697446	0.6806672	0.0903391	CCTGTTCTTGATTACTTGTTTC
mmu-miR-467c-5p	7.13E-03	0.021675393	1	1.7295242	14.536997	8.7645475	54.71416	17.898221	54.421037	16.03314	TAAGTGCGTGCATGTATATGTG
mmu-miR-378d	7.14E-03	0.021675393	1	0.1008627	1.5725206	0.670377	1.0099429	0.1594194	0.5648632	0.0993916	ACTGGACTTGGAGTCAGAAGGT
mmu-miR-425-5p	7.23E-03	0.021826036	1	0.0602518	0.8879042	0.0848391	1.3755272	0.3227571	1.4868228	0.1988631	AATGACACGATCACTCCCGTTG
oan-miR-1386	7.28E-03	0.021866895	1	0.3165177	0.6010565	0.1744502	1.1495189	0.1175332	1.4544761	0.1191957	TCGGCTCCTGGCTGGCTCGCCA
mmu-miR-126a-3p	7.49E-03	0.022391448	1	0.0215421	0.9507635	0.149753	0.7171073	0.0887186	1.0394132	0.0551625	TCGTACCGTGAGTAATAATGC
mmu-miR-143-3p	7.68E-03	0.022860619	1	0.1535765	1.365799	0.0735043	0.9270947	0.122811	0.7927775	0.1444706	TGAGATGAAGCACTGTAGCT
mmu-miR-23a-5p	7.95E-03	0.023527499	1	0.3219745	2.5127578	0.5520956	4.1096851	1.8863033	3.4214251	1.0696368	GGGGTTCCTGGGGATGGGATTT
mmu-miR-652-3p	8.19E-03	0.024134283	1	0.2428944	0.5902822	0.0065492	1.1529782	0.2409497	0.7848517	0.0659197	AATGGCGCCACTAGGGTTGTGA
mmu-miR-410-3p	8.43E-03	0.024518561	1	0.5015998	1.6942188	0.814943	0.6646572	0.1790523	2.7903132	0.6988702	AATATAACACAGATGGCCTGT
sha-miR-24	8.44E-03	0.024518561	1	0.4842972	0.4684974	0.145966	0.2489231	0.0897534	0.2578385	0.0529081	ATCTGGCTCAGTTCAGCAGGAAC
mmu-miR-182-5p	8.45E-03	0.024518561	1	0.2686847	0.7024421	0.2078117	0.4429616	0.1025267	0.3596214	0.0924438	TTTGGCAATGGTAGAACTCACACC
mmu-miR-191-3p	8.51E-03	0.024573195	1	0.4289188	1.4298961	0.2257558	2.2769702	0.5749977	2.576579	0.4763858	GCTGCACTTGGATTTCGTTC
mmu-miR-222-5p	8.73E-03	0.02508042	1	0.4709523	2.1596529	0.3088697	2.8272898	0.5811644	1.4341556	0.302107	GGCTCAGTAGCCAGTGTAGATC
mmu-miR-1948-3p	9.03E-03	0.02581485	1	0.3910427	0.4915103	0.2384145	0.6470618	0.2539182	2.4898556	0.8791734	TTTAGGCAGAGCACTCGTACAG
mmu-miR-8112	9.22E-03	0.026240084	1	0.1599005	0.5739454	0.1055694	0.9834943	0.1051947	0.8473933	0.148724	TCTCCGCCACCTCCACCGCAGC
hsa-miR-1261	9.63E-03	0.027284151	1	0.3119386	2.0406684	1.2278387	4.9569668	0.3904987	2.7144925	1.080898	TGGATAAGGCATTGGC
mmu-miR-434-3p	9.89E-03	0.027871135	1	0.2642263	2.5101291	1.2272849	0.9134584	0.1174769	1.4230546	0.1173335	TTTGAACCATCACTCGACTCCT

notes:
adj P: Benjamini-Hochberg adjusted P value.

Supplementary Table S5: 18-concurrent wound healing associated microRNA signature [a]

miRNA	tissue	p-value	adj P [b]	0 hr		6 hr		24 hr		5 day	
				fold	stdev	fold	stdev	fold	stdev	fold	stdev
mmu-miR-148b-3p	skin	1.21E-05	0.000596	1.00	0.12	1.44	0.18	3.64	0.79	2.83	0.22
	oral	3.56E-03	0.132005	1.00	0.1	1.3	0.4	2.42	0.97	3.25	0.63
mmu-miR-31-5p	skin	5.45E-05	0.001096	1.00	0.32	0.67	0.42	0.84	0.39	11.7	1.8
	oral	6.65E-03	0.151662	1.00	0.07	0.8	0.22	0.95	0.08	1.49	0.12
mmu-miR-23b-3p	skin	4.69E-03	0.016241	1.00	0.18	0.71	0.09	0.64	0.11	0.5	0.07
	oral	7.19E-03	0.151662	1.00	0.11	0.74	0.05	1.03	0.12	0.99	0.07
mmu-miR-200c-3p	skin	1.79E-03	0.008742	1.00	0.21	0.58	0.19	1.29	0.2	1.64	0.17
	oral	9.24E-03	0.163277	1.00	0.16	0.87	0.21	1.77	0.58	1.88	0.19
mmu-miR-195a-5p	skin	5.56E-05	0.001096	1.00	0.15	1.09	0.14	0.61	0.04	0.43	0.05
	oral	9.79E-03	0.163277	1.00	0.11	0.91	0.06	0.88	0.15	0.66	0.05
mmu-miR-3068-5p	skin	4.23E-05	0.001042	1.00	0.17	0.63	0.05	2.02	0.53	2.54	0.38
	oral	2.27E-04	0.075263	1.00	0.26	1.07	0.39	3.53	0.81	3.69	0.14
mmu-miR-30a-3p	skin	1.03E-04	0.001644	1.00	0.18	1.26	0.16	0.67	0.11	0.38	0.06
	oral	6.92E-04	0.086715	1.00	0.09	1.05	0.06	0.78	0.02	0.83	0.03
mmu-miR-17-5p	skin	5.92E-03	0.018935	1.00	0.33	1.23	0.09	2.46	0.42	2.19	0.7
	oral	7.15E-04	0.086715	1.00	0.16	0.85	0.05	1.51	0.16	0.99	0.05
mmu-miR-676-5p	skin	4.75E-03	0.016241	1.00	0.14	0.78	0.11	0.6	0.05	0.65	0.08
	oral	1.52E-03	0.107584	1.00	0.06	1.25	0.13	1.53	0.16	1.57	0.18
mmu-miR-362-5p	skin	4.65E-08	0.000014	1.00	0.08	0.64	0.09	4.28	0.43	5.58	0.87
	oral	2.07E-03	0.107584	1.00	0.4	0.84	0.4	3.27	1.37	4.29	0.88
mmu-miR-434-3p	skin	9.89E-03	0.027871	1.00	0.26	2.51	1.23	0.91	0.12	1.42	0.12
	oral	2.33E-03	0.111088	1.00	0.09	1.32	0.08	0.8	0.14	0.99	0.06
mmu-miR-20a-5p	skin	3.18E-03	0.012459	1.00	0.28	1.33	0.22	2.7	0.47	2.31	0.68
	oral	2.80E-03	0.123245	1.00	0.14	1.11	0.06	1.72	0.35	1.45	0.02
mmu-miR-425-3p	skin	8.08E-05	0.001402	1.00	0.24	0.61	0.18	2.12	0.1	2.23	0.22
	oral	3.55E-03	0.132006	1.00	0.15	0.8	0.34	1.59	0.01	1.91	0.13
mmu-miR-340-5p	skin	1.90E-03	0.008993	1.00	0.45	2.61	1.1	6.93	2.12	3.42	1.09
	oral	3.71E-03	0.132006	1.00	0.09	2.05	0.49	1.28	0.24	1.1	0.16
mmu-miR-664-3p	skin	2.86E-03	0.011692	1.00	0.41	1.32	0.1	0.69	0.12	0.39	0.06
	oral	4.08E-03	0.132006	1.00	0.08	0.93	0.03	0.77	0.15	0.64	0.05
mmu-miR-7a-5p	skin	5.29E-05	0.001096	1.00	0.3	1.87	0.13	5.41	0.81	5.67	1.74
	oral	4.95E-03	0.134730	1.00	0.12	1.4	0.34	2.04	0.38	1.53	0.12
mmu-miR-193b-3p	skin	2.63E-05	0.000844	1.00	0.08	0.74	0.05	0.53	0.11	0.24	0.05
	oral	5.61E-03	0.142362	1.00	0.11	0.58	0.06	0.62	0.1	0.59	0.11
mmu-miR-223-3p	skin	7.01E-05	0.001296	1.00	0.19	3.47	1.05	21.9	7.09	5.34	2.8
	oral	7.42E-03	0.151663	1.00	0.08	2.66	0.86	3.72	2.46	1.07	0.17

[a] Cut-off p-value of 0.01 was used. 18 microRNAs that were concurrently differentially expressed in both skin and oral mucosa wound healing were listed.
[b] The Benjamini-Hochberg adjusted P-values were computed for multiple hypothesis testing.

Supplementary Table S6: Expression changes of the top 10 baseline tissue specific microRNAs in skin and oral mucosa during wound healing

miR	miR-378a-3p			
time course	0hr	6hr	24hr	5day
skin (fold)	1	1.469336	0.939103	0.8457584
skin (stdev)	0.012098	0.518862	0.138471	0.087231
p value				5.26E-02
oral (fold)	0.396452	0.290532	0.378565	0.4084093
oral (stdev)	0.026353	0.013767	0.020421	0.0358472
p value				8.18E-04

miR	miR-31-5p			
time course	0hr	6hr	24hr	5day
skin (fold)	1	0.669133	0.840812	11.688945
skin (stdev)	0.320248	0.422529	0.38847	1.7979082
p value				5.45E-05
oral (fold)	22.85125	18.31651	21.60025	33.972947
oral (stdev)	1.692528	5.048494	1.765137	2.6370834
p value				6.65E-03

miR	miR-10a-5p			
time course	0hr	6hr	24hr	5day
skin (fold)	1	0.908414	0.502462	0.2383207
skin (stdev)	0.131761	0.359267	0.154867	0.0880976
p value				2.23E-03
oral (fold)	0.00482	0.006927	0.00965	0.0074661
oral (stdev)	0.002255	0.004842	0.005031	0.0046497
p value				5.64E-01

miR	miR-10b-5p			
time course	0hr	6hr	24hr	5day
skin (fold)	1	0.922428	0.302635	0.2060538
skin (stdev)	0.15904	0.40317	0.091135	0.0682445
p value				1.17E-03
oral (fold)	0.163878	0.150159	0.147039	0.1517026
oral (stdev)	0.0479	0.048789	0.039186	0.027659
p value				9.67E-01

miR	miR-133a-3p			
time course	0hr	6hr	24hr	5day
skin (fold)	1	2.0078	0.69721	0.1621058
skin (stdev)	0.565692	1.050772	0.31428	0.0153855

p value				1.11E-03
oral (fold)	0.07094	0.009774	0.014111	0.014214
oral (stdev)	0.030147	0.012295	0.012636	0.0068159
p value				6.78E-02

miR	miR-146b-5p			
time course	0hr	6hr	24hr	5day
skin (fold)	1	1.35334	1.779497	1.273712
skin (stdev)	0.098454	0.216424	0.375257	0.2865286
p value				2.65E-02
oral (fold)	0.161831	0.219098	0.364548	0.2856465
oral (stdev)	0.04531	0.07541	0.148361	0.1047957
p value				1.06E-01

miR	miR-126a-5p			
time course	0hr	6hr	24hr	5day
skin (fold)	1	1.53879	0.936007	1.5421504
skin (stdev)	0.142881	0.277857	0.32267	0.2875119
p value				4.75E-02
oral (fold)	1.864485	2.169465	1.86865	1.3459816
oral (stdev)	0.286606	0.512187	0.720687	0.2903343
p value				2.11E-01

miR	miR-34c-5p			
time course	0hr	6hr	24hr	5day
skin (fold)	1	1.890395	3.441682	35.217775
skin (stdev)	0.354481	0.313367	0.59346	29.988102
p value				1.23E-04
oral (fold)	83.6016	156.4728	64.88194	46.964317
oral (stdev)	69.09045	154.963	77.05825	36.915668
p value				6.81E-01

miR	miR-30a-5p			
time course	0hr	6hr	24hr	5day
skin (fold)	1	1.251449	0.582879	0.453159
skin (stdev)	0.103088	0.111781	0.022601	0.0342868
p value				9.89E-07
oral (fold)	0.611179	0.775242	0.580714	0.5359318
oral (stdev)	0.022063	0.210435	0.080197	0.032217
p value				9.47E-02

miR	miR-96-5p

time course	0hr	6hr	24hr	5day
skin (fold)	1	1.059792	0.343601	0.2286068
skin (stdev)	0.14515	0.384041	0.045746	0.049049
p value				1.60E-04
oral (fold)	0.577801	0.720975	0.692673	0.3836004
oral (stdev)	0.076565	0.080358	0.360337	0.0445133
p value				1.40E-01

Supplementary Table S7: Statistical analysis on TaqMan assay-based differential expression of miR-21 and miR-10l

A: Validation of differential expression in tissue samples

A.1: miR-21 in tissue samples

A.1.a: 2way ANOVA - miR-21 in tissue samples

Two-way ANOVA	Ordinary				
Alpha	0.05				
ANOVA table	SS	DF	MS	F (DFn, DFd)	P value
Interaction	51.13	3	17.04	F (3, 76) = 5.132	P = 0.0028
Row Factor (time)	156.7	3	52.22	F (3, 76) = 15.72	P < 0.0001
Column Factor (tissue type)	27.26	1	27.26	F (1, 76) = 8.209	P = 0.0054
Residual	252.4	76	3.321		

A.1.b: Multiple t tests - miR-21 in tissue samples (statistical significance determined by Holm-Sidak method)

		Mean (skin)	Mean (oral)	P value	significance	Difference	SE of diffe	t ratio	df
0 hr	Row 1	1	1.03525	0.880149		-0.0352453	0.23117	0.1525	23
6 hr	Row 2	1.10854	1.03525	0.784571		0.0732943	0.26471	0.2769	21
24 hr	Row 3	1.79576	1.09139	0.0493723	*	0.704372	0.33124	2.1265	16
5 day	Row 4	6.47986	2.49812	0.0415756	*	3.98174	1.79721	2.2155	16

A.1.c: Shapiro-Wilk tests - miR-21 in tissue samples

		skin		oral	
		P value	Null	P value	Null
0 hr	Row 1	0.427686	Accept	0.436846	Accept
6 hr	Row 2	0.090062	Accept	0.000623	Reject
24 hr	Row 3	0.569615	Accept	0.371813	Accept
5 day	Row 4	0.015668	Reject	0.145458	Accept

A.2: miR-10b in tissue samples

A.2.a: 2way ANOVA - miR-10b in tissue samples

Two-way ANOVA	Ordinary				
Alpha	0.05				
ANOVA table	SS	DF	MS	F (DFn, DFd)	P value
Interaction	2.886	3	0.962	F (3, 72) = 13.15	P < 0.0001
Row Factor (time)	2.887	3	0.9625	F (3, 72) = 13.16	P < 0.0001
Column Factor (tissue type)	7.154	1	7.154	F (1, 72) = 97.81	P < 0.0001
Residual	5.266	72	0.07314		

A.2.b: Multiple t tests - miR-10b in tissue samples (statistical significance determined by Holm-Sidak method)

		Mean (skin)	Mean (oral)	P value	significance	Difference	SE of diffe	t ratio	df
0 hr	Row 1	1	0.061636	0.000666664	*	0.938364	0.21134	4.4401	13
6 hr	Row 2	1.07812	0.047931	2.59E-07	*	1.03019	0.13262	7.7682	19
24 hr	Row 3	0.296832	0.0577083	0.00358784	*	0.239124	0.07249	3.2986	20
5 day	Row 4	0.268708	0.0485352	0.0162982	*	0.220173	0.08394	2.6229	20

A.2.c: Shapiro-Wilk tests - miR-10b in tissue samples

		skin		oral	
		P value	Null	P value	Null
0 hr	Row 1	0.23429	Accept	0.000416	Reject
6 hr	Row 2	0.988214	Accept	0.000035	Reject
24 hr	Row 3	0.008166	Reject	0.001878	Reject
5 day	Row 4	0.000341	Reject	0.004668	Reject

B: Validation of differential expression in LCM samples

B.1: miR-21 in LCM samples

B.1.a: 2way ANOVA - miR-21 in LCM samples

Two-way ANOVA	Ordinary				
Alpha	0.05				
ANOVA table	SS	DF	MS	F (DFn, DFd)	P value
Interaction	23.54	3	7.846	F (3, 24) = 7.114	P = 0.0014
Row Factor (time)	44.02	3	14.67	F (3, 24) = 13.30	P < 0.0001
Column Factor (tissue type)	23.56	1	23.56	F (1, 24) = 21.36	P = 0.0001
Residual	26.47	24	1.103		

B.1.b: Multiple t tests - miR-21 in LCM samples (statistical significance determined by Holm-Sidak method)

		Mean (skin)	Mean (oral)	P value	significance	Difference	SE of diffe	t ratio	df
0 hr	Row 1	1.1444	0.235591	0.0153373	*	0.908811	0.27094	3.3543	6
6 hr	Row 2	0.887953	0.131738	0.0163974	*	0.756214	0.22912	3.3005	6
24 hr	Row 3	1.65043	1.12877	0.530282		0.521658	0.78349	0.6658	6
5 day	Row 4	5.80185	1.12458	0.00833249	*	4.67727	1.21076	3.8631	6

B.1.c: Shapiro-Wilk tests - miR-21 in LCM samples

		skin		oral	
		P value	Null	P value	Null
0 hr	Row 1	0.947874	Accept	0.025577	Reject
6 hr	Row 2	0.75682	Accept	0.083015	Accept
24 hr	Row 3	0.147838	Accept	0.018483	Reject
5 day	Row 4	0.897928	Accept	0.344031	Accept

B.2: miR-10b in LCM samples

B.2.a: 2way ANOVA - miR-10b in LCM samples

Two-way ANOVA	Ordinary				
Alpha	0.05				
ANOVA table	SS	DF	MS	F (DFn, DFd)	P value
Interaction	1.859	3	0.6195	F (3, 24) = 7.070	P = 0.0014
Row Factor (time)	1.836	3	0.612	F (3, 24) = 6.984	P = 0.0015
Column Factor (tissue type)	4.11	1	4.11	F (1, 24) = 46.90	P < 0.0001
Residual	2.103	24	0.08763		

B.2.b: Multiple t tests - miR-10b in LCM samples (statistical significance determined by Holm-Sidak method)

		Mean (skin)	Mean (oral)	P value	significance	Difference	SE of diffe	t ratio	df
0 hr	Row 1	1.17113	0.0627785	0.00229105	*	1.10835	0.21867	5.0686	6
6 hr	Row 2	1.3093	0.028406	0.00742183	*	1.28089	0.32317	3.9635	6
24 hr	Row 3	0.310278	0.04828	0.0565111		0.261998	0.11116	2.3571	6
5 day	Row 4	0.25989	0.044054	0.0814784		0.215836	0.10322	2.0911	6

B.2.c: Shapiro-Wilk tests - miR-10b in LCM samples

		skin		oral	
		P value	Null	P value	Null
0 hr	Row 1	0.223427	Accept	0.469074	Accept
6 hr	Row 2	0.680263	Accept	0.412278	Accept
24 hr	Row 3	0.275825	Accept	0.22837	Accept
5 day	Row 4	0.048727	Reject	0.002357	Reject

Supplementary Table S8: Statistical analysis on closure of the wounds treated with miR-21 and miR-10b reagents

A: wounds treated with control and miR-21 mimic

A.1: 2way ANOVA - wounds treated with control and miR-21 mimic

Two-way ANOVA	Ordinary				
Alpha	0.05				
ANOVA table	SS	DF	MS	F (DFn, DFd)	P value
Interaction	335.2	9	37.24	F (9, 100) = 1.581	P = 0.1312
Row Factor (time)	54038	9	6004	F (9, 100) = 254.8	P < 0.0001
Column Factor (treatment)	1216	1	1216	F (1, 100) = 51.59	P < 0.0001
Residual	2356	100	23.56		

A.2: Multiple t tests - wounds treated with control and miR-21 mimic (statistical significance determined by Holm-Sidak method)

		Mean (ctrl)	Mean (miR-21)	P value	significance	Difference	SE of differ	t ratio	df
day 1	Row 1	28.5867	32.735	0.311891		-4.14833	3.89494	1.06506	10
day 2	Row 2	47.0717	51.395	0.210477		-4.32333	3.23082	1.33815	10
day 3	Row 3	54.3733	59.775	0.261995		-5.40167	4.54393	1.18876	10
day 4	Row 4	61.3967	72.4817	0.00110855	*	-11.085	2.4525	4.51988	10
day 5	Row 5	68.465	79.685	0.00036866	*	-11.22	2.13354	5.25886	10
day 6	Row 6	75.3533	84.1083	0.0126509	*	-8.755	2.88828	3.03122	10
day 7	Row 7	82.2717	90.9317	0.00292527	*	-8.66	2.21627	3.90747	10
day 8	Row 8	88.1333	94.6467	0.0391902	*	-6.51333	2.74674	2.3713	10
day 9	Row 9	95.9417	98.8817	0.00687099	*	-2.94	0.866929	3.39128	10
day 10	Row 10	98.97	99.5783	0.163996		-0.608334	0.405013	1.50201	10

A.3: Shapiro-Wilk tests - wounds treated with control and miR-21 mimic

		ctrl		miR-21	
		P value	Null	P value	Null
day 1	Row 1	0.880938	Accept	0.015552	Reject
day 2	Row 2	0.048305	Reject	0.354856	Accept
day 3	Row 3	0.965486	Accept	0.774883	Accept
day 4	Row 4	0.676812	Accept	0.591661	Accept
day 5	Row 5	0.691616	Accept	0.832817	Accept
day 6	Row 6	0.340818	Accept	0.486321	Accept
day 7	Row 7	0.065263	Accept	0.810627	Accept
day 8	Row 8	0.482778	Accept	0.409187	Accept
day 9	Row 9	0.127901	Accept	0.279401	Accept
day 10	Row 10	0.129791	Accept	0.180812	Accept

B: wounds treated with control and miR-10b LNA

B.1: 2way ANOVA - wounds treated with control and miR-10b LNA

Two-way ANOVA	Ordinary				
Alpha	0.05				
ANOVA table	SS	DF	MS	F (DFn, DFd)	P value
Interaction	529.1	9	58.79	F (9, 87) = 0.7714	P = 0.6430
Row Factor (time)	84927	9	9436	F (9, 87) = 123.8	P < 0.0001
Column Factor (treatment)	1251	1	1251	F (1, 87) = 16.42	P = 0.0001
Residual	6630	87	76.21		

B.2: Multiple t tests - wounds treated with control and miR-10b LNA (statistical significance determined by Holm-Sidak method)

		Mean (ctrl)	Mean (miR-10b)	P value	significance	Difference	SE of differ	t ratio	df
day 1	Row 1	11.2085	23.95	0.151825		-12.7414	8.13706	1.56585	9
day 2	Row 2	24.4597	33.2662	0.245699		-8.80655	7.09181	1.24179	9
day 3	Row 3	36.2235	48.8293	0.100135		-12.6058	6.87992	1.83227	9
day 4	Row 4	47.6599	52.779	0.434157		-5.11904	6.25346	0.818593	9
day 5	Row 5	54.4978	60.861	0.283508		-6.36319	5.57918	1.14052	9
day 6	Row 6	63.9945	70.4322	0.279583		-6.43774	5.41722	1.18838	6
day 7	Row 7	74.8959	87.2205	0.00296294	*	-12.3246	3.0565	4.03225	9
day 8	Row 8	93.4787	97.2499	0.296344		-3.77115	3.4017	1.10861	9
day 9	Row 9	97.7562	98.1437	0.77031		-0.387475	1.28763	0.300922	9
day 10	Row 10	99.302	100	0.00186593		-0.69804	0.160682	4.34422	9

B.3: Shapiro-Wilk tests - wounds treated with control and miR-10b LNA

		ctrl		miR-10b	
		P value	Null	P value	Null
day 1	Row 1	0.079956	Accept	0.829051	Accept
day 2	Row 2	0.230435	Accept	0.450255	Accept
day 3	Row 3	0.096018	Accept	0.758435	Accept
day 4	Row 4	0.081085	Accept	0.355892	Accept
day 5	Row 5	0.240936	Accept	0.131831	Accept
day 6	Row 6	0.495443	Accept	0.904362	Accept
day 7	Row 7	0.659012	Accept	0.248763	Accept
day 8	Row 8	0.016694	Reject	0.050619	Accept
day 9	Row 9	0.907885	Accept	0.017018	Reject
day 10	Row 10	0.565286	Accept	NaN	Reject
